# Association of sugary beverages consumption with liver fat content and fibro-inflammation: a large cohort study

**DOI:** 10.3389/fpubh.2025.1624848

**Published:** 2025-10-09

**Authors:** Yiheng Zhou, Yu Jia, Yi Yao, Yu Cheng, Yonglang Cheng, Rong Yang, Rui Zeng, Zhi Wan, Qian Zhao, Dongze Li, Yi Lei, Xiaoyang Liao

**Affiliations:** ^1^General Practice Ward/International Medical Center Ward, General Practice Medical Center, West China Hospital, Sichuan University, Chengdu, China; ^2^Department of Cardiology, West China Hospital, West China School of Medicine, Sichuan University, Chengdu, Sichuan, China; ^3^Department of Emergency Medicine, Disaster Medical Center, West China Hospital, West China School of Medicine, Sichuan University, Chengdu, Sichuan, China

**Keywords:** sugary beverages, liver fat content, hepatic fibro-inflammation, magnetic resonance imaging, proton density fat fraction

## Abstract

**Objective:**

Liver fat content (LFC) and hepatic fibro-inflammation (HFI) accumulation are the primary pathological manifestation of steatohepatitis. The association between intake of sugary beverages (SBs), including artificially-sweetened beverages (ASB), sugar-sweetened beverages (SSB), and natural juices (NJs), and LFC or HFI remains unclear.

**Methods:**

The study included 25,885 participant who completed at least one online dietary assessment and magnetic resonance imaging. LFC and HFI were quantified using the liver proton density fat fraction (PDFF) and iron-corrected T1 (cT1).

**Results:**

Compared to those without ASB and SSB intake, the arithmetic mean difference (AMD) of PDFF was 0.15 (95% Cl: 0.06 to 0.24) and 0.21 (95% Cl, 0.12 to 0.29), and the AMD of cT1 was 3.86 (95% CI, 1.26 to 6.79) and 2.43 (95% CI, 1.31 to 3.57) in individuals with ≥1 serving/d, respectively. Individuals with 0–1 serving/d had lower PDFF (AMD: −0.10 95%Cl: −0.19 to −0.01) than those without NJs intake. In Quantile G-computation models, SSB and ASB contributed most in the AMD of PDFF (54.7%) and cT1 (53.1%), respectively. When replacing ASB and SSB with water, the progress of PDFF was improved.

**Conclusion:**

Artificially-sweetened beverages and SSB intake were positively associated with LFC and HFI, and moderate NJs intake was slightly negatively associated with LFC but not HFI.

## Introduction

Liver fat content (LFC) is a hallmark of metabolic dysfunction-associated steatotic liver disease (MASLD), affecting over 30% of the global population and imposing a significant economic burden (1, 2). While hepatic fibro-inflammation (HFI) reflects the severity of MASLD, excessive LFC accumulation is a precursor to HFI, which further leads to liver cirrhosis, liver failure, and hepatocellular carcinoma (3). Moreover, studies reported that elevated LFC and HFI exacerbate the progression of extrahepatic diseases including diabetes, hypertension, and other cardiometabolic conditions (4, 5). Despite the need for effective interventions, there is a paucity of medications to reduce LFC and HFI, making diet intervention a cornerstone of current guidelines (5, 6). However, as a crucial part of dietary interventions, the relationship between LFC, HFI, and sugary beverages (SBs), including artificially-sweetened beverages (ASB), sugar-sweetened beverages (SSB), and natural juices (NJs), remains unclear.

Previous research and guidelines have highlighted the detrimental effects of SSB on LFC (7, 8). Yet, the association between ASB and NJs intake and LFC development is not well-established. For example, two randomized controlled trials have indicated that replacing SSB with ASB might decrease LFC (9, 10), suggesting that ASB could serve as a potential substitute for SSB due to their lower calorie and sugar content (11). However, these studies were limited by short follow-up periods, small sample sizes, and a focus on overweight and obese populations. Additionally, ASB have been shown to negatively affect intestinal microecology, glucose homeostasis, adipose tissue deposition, weight gain, and metabolic syndrome (12, 13). Moreover, numerous studies have also linked ASB consumption to cardiometabolic diseases, including type 2 diabetes, cardiovascular diseases, thrombosis, and mortality (14–16). Thus, caution should be exercised when considering ASB as a substitute for SSB in managing steatotic liver disease (SLD) and non-alcoholic steatohepatitis (NASH). Natural juices, another category of SBs, contain both harmful and beneficial bioactive molecules like fructose, micronutrients, and antioxidants, potentially impacting LFC development (17, 18). However, few studies have explored the relationship between NJs intake and LFC. Moreover, to our knowledge, there have been no studies that have yet explored the relationship between sugary beverages (SBs) and hepatic fibro-inflammation (HFI) levels. Therefore, further research with long-term follow-up and large populations is necessary to elucidate these associations.

Consequently, our study aimed to investigate the association between SBs intake (ASB, SSB, and NJs), LFC, and HFI, as well as to assess their joint associations and relative importance based on a large community-based cohort with long-term follow-up. Additionally, the study explores the effects of beverage substitution and potential mediators on these associations.

## Materials and methods

### Study design and population

The data originated from the UK Biobank, an extensive, prospective cohort study encompassing more than 500,000 participants within the age range of 37–63 years. Affirming their voluntary participation, the individuals provided written consent via electronic questionnaires for the collection of their data. The study incorporated comprehensive magnetic resonance imaging (MRI) derived from a multimodal imaging initiative, yielding a rich dataset of imaging information. Adhering to stringent ethical research standards, the UK Biobank study has received clearance from the Northwest Multicenter Research Ethics Committee. Authorization for the utilization of this dataset has also been granted by the Human Ethics Committee of West China Hospital, Sichuan University.

**Figure 1:**
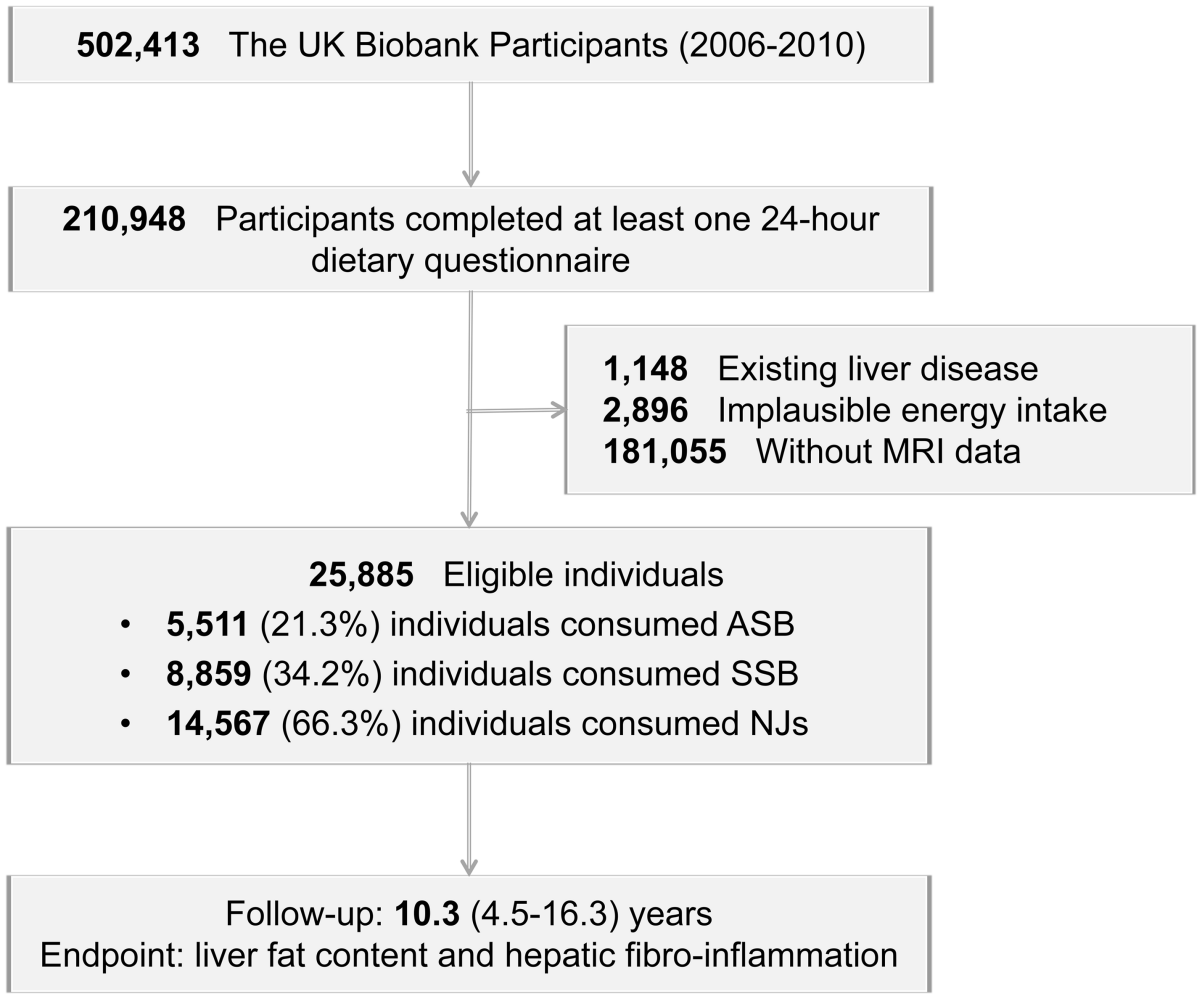
Study flow chart.

A total of 210,948 participants completed at least one 24-h dietary recall. Participants were excluded if they had existing liver disease (Supplementary Table S1, *n* = 1,148), implausible energy intake levels (women: <500 or >3,500 kcal/day; men: <800 or >4,000 kcal/day, *n* = 2,896), or lacked MRI data (*n* = 181,055). Following these exclusions, the study included 25,885 eligible individuals (Figure 1).

### Assessment of intake of beverages

Participants were asked to complete online 24-h dietary assessments, providing detailed recalls of their consumption of 206 common foods and 32 beverages over the preceding 24 h. Daily beverage intake was assessed by following question: “How many glasses, cans, or cartons containing 250 mL of sugar-sweetened beverages, artificially sweetened beverages, or natural juices did you drink yesterday?” Participants were categorized into 3 groups based on the distribution of these products: 0, 0 to 1 and ≥1 serving per day. From 2009 to 2012, participants were asked online five times, and those who completed at least one dietary assessment were included in the study by calculating their mean beverage intake. The beverages intake levels were comparable with the national data in the UK (19).

### Assessment of liver fat content and hepatic fibro-inflammation

The Proton Density Fat Fraction (PDFF), ascertained through Magnetic Resonance Imaging (MRI), represents the proportion of protons associated with fat relative to the overall proton count within the liver. This method allows for the direct measurement of LFC, eliminating the need for invasive biopsy, which was considered as the most accurate noninvasive method (20). Previous study showed MRI was an excellent assessment compared with liver biopsy (Spearman correlation coefficient = 0.85) (21) and can classify grades of hepatic steatosis with areas under the summary receiver operating characteristic curves ≥90% (22). A cardiac-gated Shortened Modified Look-Locker Inversion sequence was utilized to measure liver T1 values, and it can be adjusted to account for the influence of iron, resulting in an cT1 score, which is expressed in milliseconds (ms) and serves as an indirect indicator of hepatic fibro-inflammatory activity. This metric, derived from MRI, has been confirmed for accuracy by comparison with liver histology and has shown its practical use in clinical settings going forward (23–26).

### Covariates assessment

Our models were adjusted for several covariates: sex (male and female), age, Townsend deprivation index, education statues, alcohol intake (g/d), smoking status (never, current, former), BMI (<25.0, 25.0 to <30, ≥30 kg/m^2^), abdominal obesity (yes and no), physical activity (metabolic equivalents [MET] hours per day for all physical activity), hypertension (yes and no), glucose (mmol/L), triglyceride (mmol/L), cholesterol (mmol/L), C-reactive protein (mg/L), and platelet distribution width (%), total energy (kcal/d), total sugar (g/d), and healthy diet score (0–7). Healthy diet score were based on following criterion (27): total vegetables, ≥4 servings per day; total fruit, ≥4 servings per day; total fish, ≥2 servings per week; processed meat, ≤1 servings per week; red meat, ≤1.5 servings per week; whole grains, ≥3 servings per day; refined grains, ≤1.5 servings per day and achieving one of the above criteria is scored as one point (ranged from 0 to 7). Covariates were collected by professionals through questionnaires, physical examinations, and biological samples (28). The detailed descriptions of covariates were performed in Supplementary material according to previous research (27).

### Statistical analysis

Continuous variables were presented as means with standard deviations and categorical variables were depicted as counts and percentages. According to previous study (29), we performed univariate and multivariate linear regression models and calculated the arithmetic mean difference (AMD) and 95% confidence intervals (CIs) to explore the correlation PDFF, cT1, and SBs consumption. In addition, we defined hepatic steatosis as PDFF ≥ 5% (30) and hepatitis as cT1 ≥ 800 ms (31), and investigated their associations with beverage intake. Restricted cubic splines with three knots (knots placed at the quartile of each beverage intake) were used in unadjusted and fully adjusted models to explore the dose–response relationships between SBs intake, PDFF, and cT1, respectively. Additionally, subgroup analyses were conducted to explore the relationship of AMD of PDFF and cT1 with SBs intake across different subgroups, categorized by age (<55 vs. ≥55), sex (male vs. ≥female), alcohol intake (<10 g/d vs. ≥10 g/d), BMI (<25.0 vs. 25.0 to <30 vs. ≥30 kg/m^2^) physical activity (whether met WHO physical activity guidelines: 150 min of moderate activity per week or 75 min of vigorous activity) (32). Quantile G-computation (QGC) (33) model was employed to comprehensively evaluate the relative importance and joint association of various beverage intakes on Proton Density Fat Fraction (PDFF) and contrast-enhanced T1-weighted imaging (cT1) outcomes. The dietary questionnaire inquires about the servings of beverages consumed within 24 h (250 mL = 1 serving). Therefore, we utilized substitution models to assess the effect of replacing one serving of a beverage with another (34). Moderation analysis was conducted to investigate which covariates moderated the effect of ASB and SSB intake on PDFF and cT1. We also performed a mediation analysis to quantify the proportion of PDFF and cT1 explained by indirect factors as well as the direct association of SSB intake and ASB intake. The detailed descriptions of statistical analysis were provided in Supplementary material.

To robust our findings, several sensitivity analyses were conducted. First, we divided health diet score into the original seven parts for adjustment. Second, we additionally adjusted for carbohydrates intake, use of medication (aspirin, cholesterol lowering, hypoglycemic, and antihypertensive medication) due to potential confounding. Third, the percentage change of PDFF also expressed by linear regression models. Fourth, the linear regression models was repeated in participants who completed at least 2 dietary assessments.

Statistical significance was set at a two-tailed *p*-value of <0.05. All statistical analyses were performed using SPSS (version 27.0; IBM Corp., Armonk, NY, United States) and R software 3.5.0 (Vienna, Austria).

## Results

### Baseline characteristics

A total of 25,885 participants (age: 55.3 ± 7.5 years, male: 12,228 [47.2%]) were included in our study with a median follow-up of 10.3 (4.5–16.3) years. Among these population, 5,511 (21.3%) individuals consumed ASB, 8859 (34.2%) individuals consumed SSB, and 14,567 (66.3%) individuals consumed NJs. Compared to those without SBs intake, participants with ≥1 serving/d more likely to be younger, current smoker and had higher BMI and energy intake and less physical activity. For biochemical examination, they had higher glucose, triglyceride, cholesterol, liver enzyme (Table 1 and Supplementary Table S2).

**Table 1:** Baseline characteristics of participants by sugar-sweetened beverages intake.

Characteristics	Sugar-sweetened beverages			*p*-value
	0/d	0–1/d	≥1/d	
Sample size, *n*(%)	17,026 (65.8%)	1,603 (6.3%)	7,202 (27.8%)	
Male, *n*(%)	7,803 (45.8%)	777 (47.7%)	3,648 (50.7%)	<0.001
Age (years)	55.36 (7.41)	55.28 (7.63)	53.44 (7.59)	<0.001
Townsend deprivation Index	−1.90 (2.72)	−1.88 (2.66)	−1.80 (2.74)	0.819
Education				<0.001
College or University degree	8,817 (51.8%)	766 (47.0%)	3,567 (49.5%)	
A AS level or equivalent	2,225 (13.1%)	217 (13.3%)	992 (13.8%)	
O levels or equivalent	3,510 (20.6%)	371 (22.8%)	1,630 (22.6%)	
Other	2,474 (14.5%)	276 (16.9%)	1,013 (14.1%)	
Alcohol intake (g/d)	11.36 (9.81)	10.20 (9.12)	10.80 (10.54)	<0.001
Smoking status, *n*(%)				<0.001
Never	10,248 (60.2%)	1,038 (63.7%)	4,552 (63.2%)	
Former	995 (5.8%)	88 (5.4%)	432 (6.0%)	
Current	5,783 (34.0%)	504 (30.9%)	2,218 (30.8%)	
Abdominal obesity, *n*(%)	3,925 (23.1%)	366 (22.5%)	1822 (25.3%)	<0.001
BMI (kg/m^2^)	26.36 (4.02)	26.49 (4.02)	26.91 (4.26)	<0.001
Physical activity (MET hours/week)	40.36 (36.25)	39.73 (35.74)	39.51 (39.11)	<0.001
Hypertension, *n*(%)	6,662 (39.1%)	655 (40.2%)	2,704 (37.5%)	0.033
Diabetes, *n*(%)	503 (3.0%)	34 (2.1%)	139 (1.9%)	<0.001
Albumin (g/L)	45.36 (2.52)	45.43 (2.53)	45.52 (2.52)	<0.001
Glucose (mmol/L)	5.36 (0.97)	4.99 (0.93)	5.42 (0.91)	<0.001
Triglyceride (mmol/L)	1.36 (0.91)	1.63 (0.91)	1.76 (1.06)	<0.001
Cholesterol (mmol/L)	5.36 (1.10)	5.75 (1.10)	5.65 (1.07)	0.304
C-reactive protein (mg/L)	1.36 (3.27)	2.07 (3.90)	2.18 (3.80)	<0.001
Platelet count (10^9/^L)	248.9 (64.54)	247.8 (65.15)	250.9 (65.37)	<0.001
HDL-c (mmol/L)	1.36 (0.38)	1.47 (0.37)	1.42 (0.36)	<0.001
LDL-c (mmol/L)	3.36 (0.84)	3.61 (0.83)	3.55 (0.81)	<0.001
ALT (U/L)	22.36 (12.42)	22.62 (14.08)	23.79 (15.64)	<0.001
AST (U/L)	25.36 (7.83)	25.56 (10.72)	26.27 (10.47)	<0.001
GGT (U/L)	31.36 (32.93)	32.31 (28.60)	33.78 (28.70)	<0.001
Energy (kJ/d)	8520.36 (2026.19)	8821.54 (2020.28)	9296.56 (2080.84)	<0.001
Sugar intake (g/d)	117.36 (38.52)	129.53 (39.53)	148.02 (43.37)	<0.001
Healthy diet score	3.36 (1.34)	2.98 (1.35)	2.91 (1.41)	<0.001

BMI, body mass index; HDL-c, high-density lipoprotein cholesterol; LDL-c, low-density lipoprotein cholesterol; ALT, alkaline phosphatase; AST, glutamic oxaloacetic transaminase; GGT, glutamyl transpeptidase; MET, metabolic equivalent.

### Linear and logistic regression analysis and restricted cubic splines

In the fully adjusted linear regression models (Table 2 and Supplementary Tables S3, S4), compared to those without ASB and SSB intake, the arithmetic mean difference (AMD) of PDFF was 0.15 (95% CI, 0.06 to 0.24, *p* < 0.001) and 0.21 (95% CI: 0.12 to 0.29, *p* < 0.001), and the AMD of cT1 was 3.86 (95% CI, 1.26 to 6.79, *p* < 0.001) and 2.43 (95% CI: 1.31 to 3.57, *p* < 0.001) in individuals with ≥1 serving/d, respectively. Additionally, there were both higher PDFF and cT1 for each additional serving of ASB and SSB intake. Compared to non-NJs intake population, those with 0–1 serving/d had decreased PDFF (AMD: −0.10, 95%CI: −0.19 to −0.01). However, this association was not significant between NJs and cT1. In addition, using PDFF ≥ 5% as the threshold for hepatic steatosis and cT1 ≥ 800 ms as the threshold for hepatitis, we found that compared to those without ASB and SSB intake, the odds ratio of hepatic steatosis was 1.08 (95% CI, 1.03 to 1.15, *p* = 0.05) and 1.14 (95% CI: 1.02 to 1.23, *p* = 0.008) and the OR of hepatitis was 1.33 (95% CI, 1.10 to 1.65, *p* < 0.001) and 1.29 (95% CI: 1.12 to 1.48, *p* < 0.001) in individuals with ≥1 serving/d, respectively (Supplementary Table S5).

**Table 2:** Linear regression models were performed to analyze the association between category of beverages intake and PDFF as well as cT1.

Category of beverage intake	PDFF				cT1			
	Unadjusted difference (95%Cl)	*P*	Adjusted difference (95%Cl)	*P*	Unadjusted difference (95%Cl)	*P*	Adjusted difference (95%Cl)	*P*
Artificially-sweetened beverage								
0 serving/d	reference		reference		reference		reference	
0–1 serving/d	0.36 (0.10, 0.63)	0.006	−0.04 (−0.24, 0.17)	0.696	8.48 (6.63, 10.34)	<0.001	2.48 (0.75, 4.21)	0.005
≥1 serving/d	0.62 (0.51, 0.74)	<0.001	0.15 (0.06, 0.24)	<0.001	9.32 (4.82, 13.82)	<0.001	3.86 (1.26, 6.79)	<0.001
Per 1 serving/d increased	0.33 (0.27, 0.39)	<0.001	0.07 (0.02, 0.12)	0.003	5.06 (4.08, 6.05)	<0.001	1.67 (0.76, 2.59)	<0.001
Sugar-sweetened beverages								
0 serving/d	reference		reference		reference		reference	
0–1 serving/d	0.18 (0.01, 0.35)	0.037	0.19 (0.05, 0.34)	0.007	5.80 (2.83, 8.77)	<0.001	1.94 (0.40, 3.49)	0.014
≥1 serving/d	0.38 (0.29, 0.48)	<0.001	0.21 (0.12, 0.29)	<0.001	6.34 (4.70, 7.97)	<0.001	2.43 (1.31, 3.57)	<0.001
Per 1 serving/d increased	0.26 (0.20, 0.31)	<0.001	0.12 (0.07, 0.16)	<0.001	3.59 (3.01, 4.90)	<0.001	1.06 (0.35, 1.97)	0.007
Nature juices								
0 serving/d	reference		reference		reference		reference	
0–1 serving/d	−0.30 (−0.40, −0.19)	<0.001	−0.10 (−0.19, −0.01)	0.027	−2.03 (−3.88, −0.19)	0.031	−0.14 (−1.86, 1.56)	0.866
≥1 serving/d	−0.14 (−0.23, −0.04)	0.007	0.01 (−0.07, 0.10)	0.706	0.35 (−1.34, 2.04)	0.684	0.77 (−0.84, 2.38)	0.394
Per 1 serving/d increased	−0.05 (−0.13, 0.02)	0.150	0.03 (−0.03, 0.09)	0.348	1.27 (0.02, 2.57)	0.045	0.94 (−0.23, 2.12)	0.116

Models were adjusted for age, sex, Deprivation Index, education, alcohol intake, smoking status, hypertension, diabetes, physical activity, laboratory measurements (glucose, triglyceride, cholesterol, C-reactive protein and platelet distribution width), dietary intake (total energy, total sugar, and healthy diet score), body mass index and abdominal obesity.

**Figure 2:**
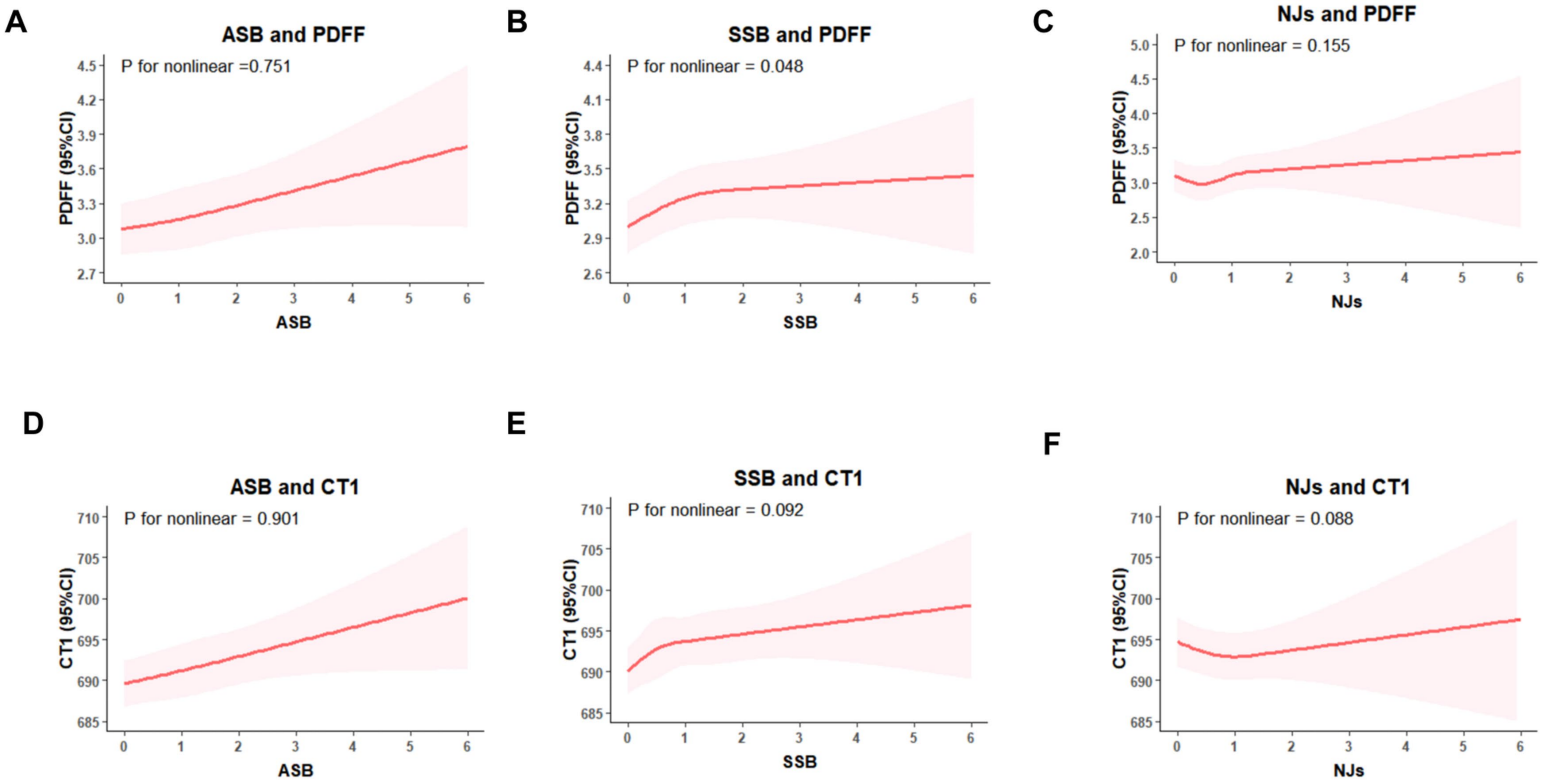
Restricted cubic splines models for the association between sugary beverages intake, PDFF, and cT1. **(A)** ASB intake and PDFF, **(B)** SSB intake and PDFF, **(C)** NJs intake and PDFF; **(D)** ASB intake and cT1; **(E)** SSB intake and cT1; **(F)** NJs intake and cT1. Models were adjusted for age, sex, Deprivation Index, education, alcohol intake, smoking status, physical activity, hypertension, diabetes, laboratory measurements (glucose, triglyceride, cholesterol, C-reactive protein and platelet distribution width), dietary intake (total energy, total sugar, and healthy diet score), body mass index and abdominal obesity.

In fully adjusted restricted cubic splines (Figure 2), ASB intake showed linear dose–response association with PDFF and cT1, and there was a non-linear dose–response association of SSB with PDFF (*P* for nonlinear = 0.048), with PDFF plateauing when SSB intake reaches 2 servings/day. However, significant dose–response association between NJs intake, PDFF, and cT1 were not observed (*P* for overall >0.05).

### Joint and substitution association analyses

**Figure 3:**
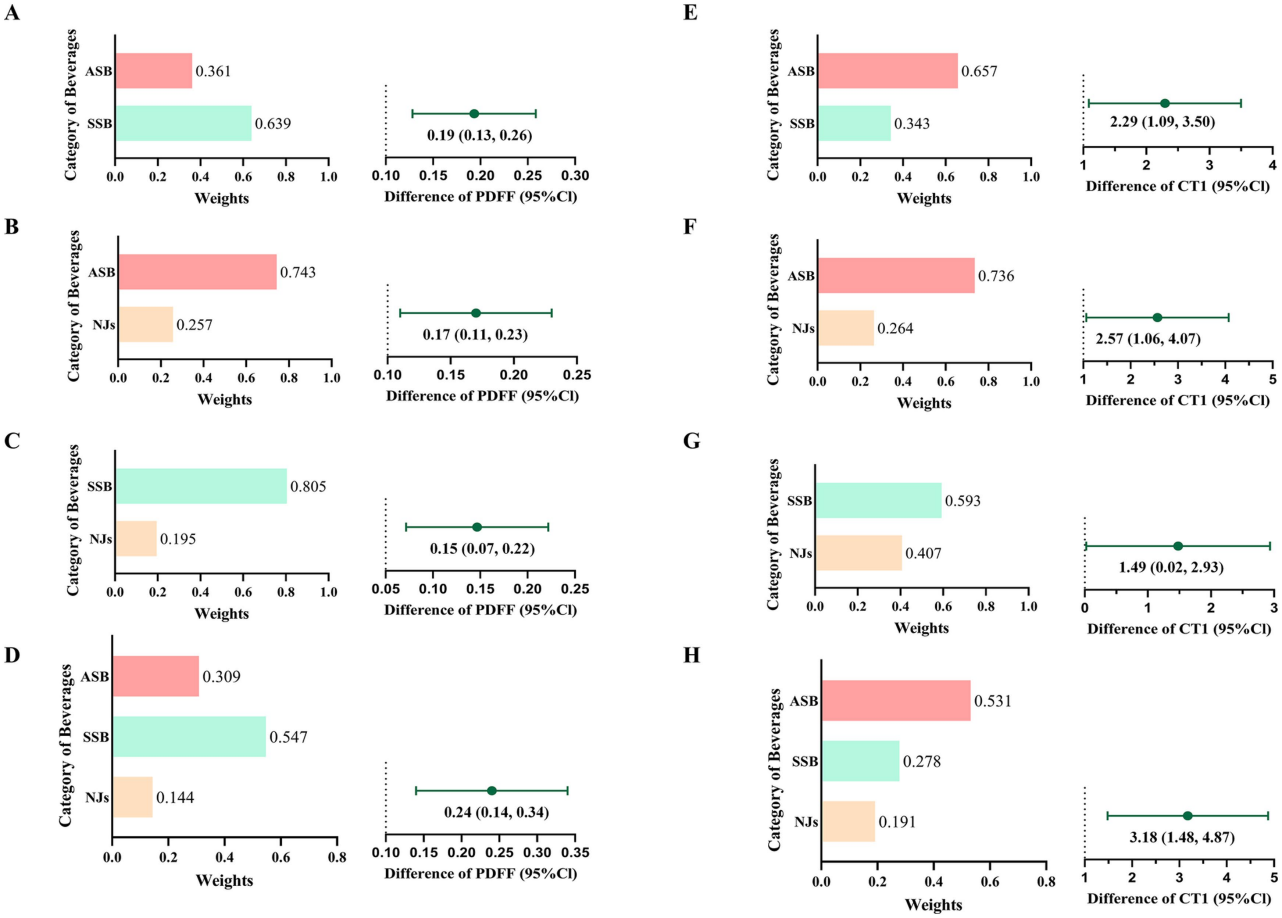
Joint associations and relative contributions of sugary beverages to PDFF **(A–D)** and cT1 **(E–H)**. A/E): joint associations and relative contributions of ASB and SSB; B/F): joint associations and relative contributions of ASB and NJs; C/G): joint associations and relative contributions of SSB and NJs; D/H): joint associations and relative contributions of ASB, SSB and NJs.

In fully adjusted QGC models (Figure 3), for each extra serving of SBs intake, the AMD of PDFF was 0.20 (95%CI: 0.12 to 0.28), and SSB contributed the most (54.7%); While, the AMD of cT1 was 3.18 (95%CI: 1.48 to 4.87), and ASB contributed the most (53.1%).

In substitution analysis (Table 3), the PDFF was decreased when substituting SSB with NJs and water, and substituting ASB with water. Moreover, a decrease in cT1 (AMD: −1.78, 95%CI: −2.76 to −0.80) was observed after substituting ASB with water.

**Table 3:** Substitution analysis examining the association between PDFF as well as cT1 and category of beverage intake.

Substitution analysis	ASB		SSB		NJs		Water	
	Difference (95%Cl)	*P*	Difference (95%Cl)	*P*	Difference (95%Cl)	*P*	Difference (95%Cl)	*P*
PDFF								
With ASB	reference	–	−0.05 (−0.12, 0.02)	0.177	0.05 (−0.13, 0.02)	0.179	0.06 (0.01, 0.12)	**0.014**
With SSB	0.05 (−0.02, 0.12)	0.177	reference	–	0.09 (0.02, 0.17)	**0.016**	0.11 (0.06, 0.16)	**<0.001**
With NJs	−0.05 (−0.02, 0.13)	0.179	−0.09 (−0.17, −0.02)	**0.016**	reference	–	−0.01 (−0.07, 0.05)	0.775
With water	−0.06 (−0.12, −0.01)	**0.014**	−0.11 (−0.16, −0.06)	**<0.001**	0.01 (−0.05, 0.07)	0.775	reference	–
CT1								
With ASB	reference	–	1.08 (−0.31, 2.48)	0.126	0.90 (−0.59, 2.39)	0.235	1.78 (0.80, 2.76)	**<0.001**
With SSB	−1.08 (−2.48, 0.31)	0.126	reference	–	0.03 (−1.45, 1.50)	0.973	0.83 (−0.12, 1.79)	0.087
With NJs	−0.90 (−2.39, 0.59)	0.235	−0.03 (−1.50, 1.45)	0.973	reference	–	0.86 (−0.37, 2.09)	0.171
With water	−1.78 (−2.76, −0.80)	**<0.001**	−0.83 (−1.79, 0.12)	0.087	−0.86 (−2.09, 0.37)	0.171	reference	–

ASB, artificially-sweetened beverage; SSB, sugar-sweetened beverages; NJs, nature juices. Models were adjusted for age, sex, Deprivation Index, education, alcohol intake, smoking status, physical activity, hypertension, diabetes, laboratory measurements (glucose, triglyceride, cholesterol, C-reactive protein and platelet distribution width), dietary intake (total energy, total sugar, and healthy diet score), body mass index and abdominal obesity. Bold figures denote *P* < 0.05.

### Subgroup, moderation, mediation and sensitivity analyses

In subgroup analyses (Supplementary Tables S6–S10), the associations of PDFF, cT1, and SBs intake were broadly similar in different subgroups of sex, age, BMI, and physical activity (*P* for interaction >0.05). However, there was a stronger association among those who consumed more alcohol (*P* for interaction = 0.002). In moderation analysis (Supplementary Tables S11, S12), we found alcohol intake (ASB: *P* for interaction = 0.002; SSB: *P* for interaction <0.001) and sugar intake (ASB: *P* for interaction = 0.009; SSB: *P* for interaction = 0.004) had a positive moderating effect (*β* > 0) on the association of ASB and SSB consumption with PDFF. Moreover, abdominal obesity was also a significant moderate factor for cT1.

**Figure 4:**
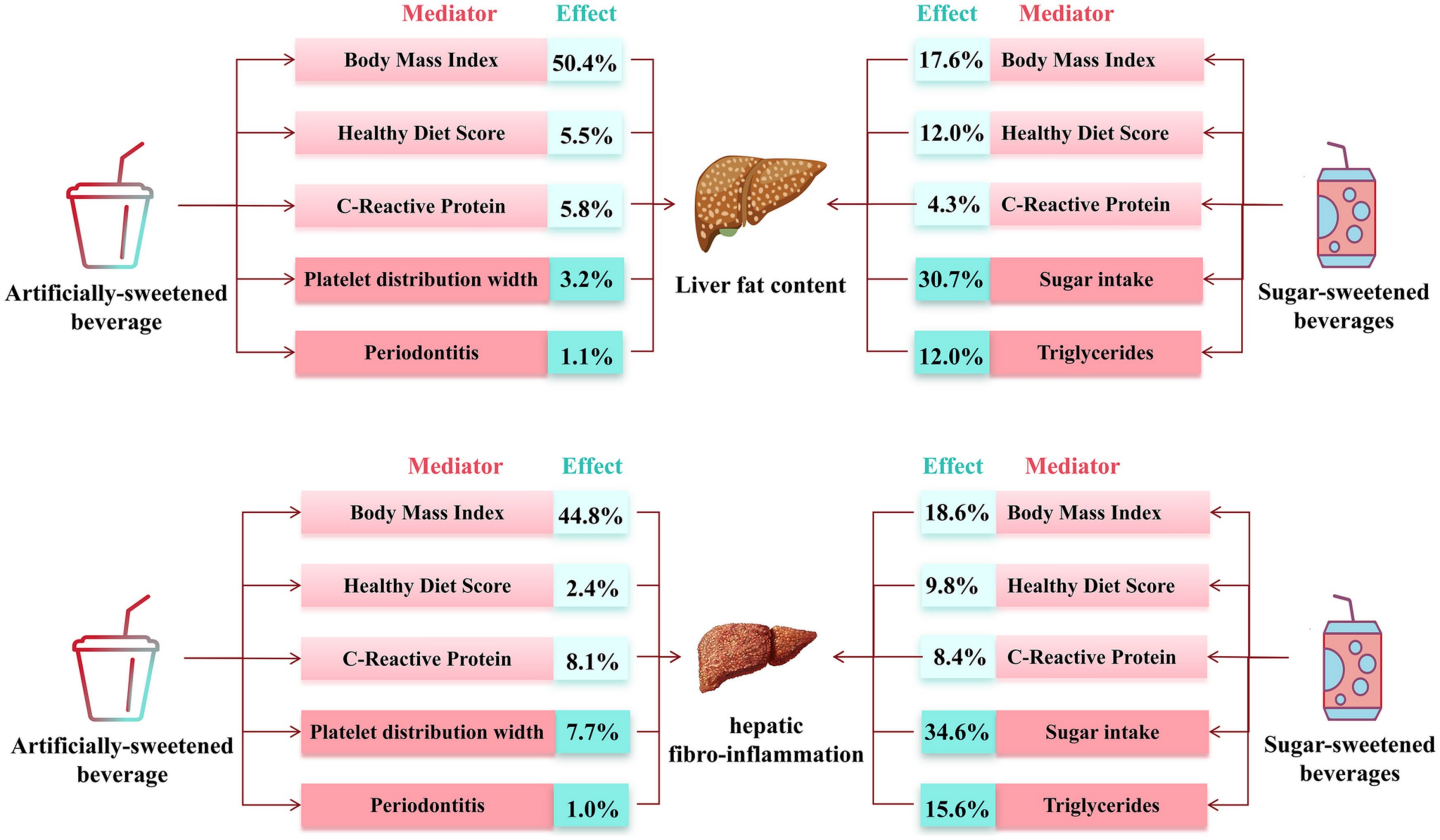
Estimated direct and indirect effect category of beverage intake on PDFF and cT1 mediated by mediators.

In mediation analyses (Figure 4 and Supplementary Tables S13, S14), body fat, healthy diet, and inflammation showed an partial mediation effect on the association of ASB and SSB consumption with PDFF and cT1. On the other hand, sugar intake and triglycerides also served as an adverse mediated factors for SSB. Considering that there was no dose–response association between NJs intake, PDFF, and cT1, the mediation analysis did not conducted for NJs.

In sensitivity analyses (Supplementary Tables S15–S18), the associations remained robust after adjusting additional covariates, including original seven parts of healthy diet score, carbohydrate intake, aspirin, and lowering cholesterol, hypoglycemic, and antihypertensive medication; including population completed at least 2 dietary questionnaires; and representing difference as percentage change of PDFF.

## Discussion

To best of our knowledge, this is the first study to investigate the relationship between beverage consumption, LFC, and HFI with community-based design and MRI measurement. In our research, population with ≥1 serving/d of ASB and SSB had higher LFC and HFI than those without ASB or SSB intake. Furthermore, ASB and SSB consumption showed a positive dose–response association with LFC. Notably, moderate NJs intake (0–1 serving per day) was inversely with LFC, but this association not remain significant for HFI. Our study provided novel evidence for the prevention of SLD and indicated that management of beverage intake may be a useful approach to preventing chronic liver disease.

The adverse impact of SSB and ASB intake on LFC is consistent in different sex, age, BMI and physical activity level. However, individuals with higher alcohol and sugar intake may be more susceptible to LFC development when consuming ASB and SSB. BMI, healthy diet score and C-reaction protein both partly mediated the association between beverage intake and LFC. Furthermore, platelet function and periodontitis may be potential reasons for association between ASB intake, LFC, and HFI. It indicated that, although ASB can reduce sugar and calorie intake, they can still exacerbate liver pathological changes through other pathways, such as inflammation, platelet function, and an unhealthy diet. Which is consistent with previous studies (16, 35). Contrary to previous research, we found that substituting SSB with ASB was not associated with reduced LFC or HFI, indicating that ASB is not an appropriate alternative to SSB. Moreover, only water can be reliable replacement to reduce the impact of ASB or SSB on LFC or HFI.

Our findings can be supported by several previous studies. A prospective study showed that more ASB consumption was associated with higher incidence of MASLD and moderated NJs consumption was not associated with lower incidence of MASLD (36). Many studies found the positive association between SSB intake and fatty liver disease (7, 8, 37). However, a Framingham Heart Study cohorts study revealed that diet soda intake (a type of ASB) was not positively related with risk of fatty liver disease (8). Two small clinical intervention studies (*n* = 47, *n* = 27) and a meta-analysis showed that replacing SSB with ASB would decrease deposition of fat in liver (9–11). These contradictory results may contribute to differences in experimental design, heterogeneity of population, sample sizes, assessment of beverage intake, and measurement of LFC. Notably, evidence regarding the relationship between SBs and HFI is lack. Therefore, our study is the first to reveal the long-term adverse impact of ASB and SSB intake on LFC and HFI development with large population.

Although the mechanism of the relationship between beverages intake and liver histology is not fully clarified, several reasons may be explained. First, SSB is a major contributor to free sugar intake, which can lead to increased calorie consumption, high glycemic load, elevated blood glucose, hyperinsulinemia, and insulin resistance, thereby increasing LFC (38, 39). Second, in animal models, artificial sweeteners have adverse influence on component and function of host intestinal microecology, glucose homeostasis, inflammation and adipose tissue deposition. In addition, evidence from human studies associates the consumption of artificial sweeteners with weight gain and metabolic syndrome (12, 13). Third, for NJs, on the one hand, more natural sugar (such as fructose) may increase the lipid accumulation in liver by promoting the expression of fatty acid synthase (40, 41). On the other hand, NJs have a large content of bioactive molecules, such as vitamin C (17), carotenoids (18) and flavonoids (42), potentially lowering inflammation and oxidative stress in liver and then inhibit the pathological progression. Further study is urgently needed to clarify the mechanism.

Our study has several limitations. First, as a prospective cohort study, this analysis cannot establish a causal relationship between beverage intake and LFC or HFI. Second, although dietary questionnaires were collected on five separate occasions, this recall-based method is inevitably subject to recall bias, which may lead to either an over- or underestimation of habitual intake for certain foods. Moreover, the population is mainly aged over 50 years and caucasians, which may restrict the generalizability of our findings. Previous studies have shown that food preferences, nutrient metabolism, and disease susceptibility vary by age and ethnicity (43, 44), which may alter the strength or even the direction of the diet–disease associations observed here. Therefore, whether these conclusions apply to younger individuals or to other ethnic groups remains to be verified. Forth, the baseline of MRI measurements is lack in database so that we cannot assess changes in LFC or HFI over time; only a single measurement is available as the outcome. Furthermore, although our models adjusted for multiple covariates, residual confounding from incompletely controlled factors—such as insulin resistance, socioeconomic status, and types of artificial sweeteners—may still bias the results.

## Conclusion

In conclusion, ASB and SSB intake were positively associated with LFC and HFI, while moderate NJs intake was inversely associated with LFC, but not HFI. The combined consumption of SBs was associated with development of LFC and HFI, and ASB intake showed a stronger positive association with HFI progression than SSB intake. Moreover, replacing ASB with water was associated with protective effect on both LFC and HFI. Overall, our findings support substitution strategies as a potential approach, but randomized controlled intervention trials are still needed to confirm its efficacy and safety.

## Data Availability

The datasets presented in this study can be found in online repositories. The names of the repository/repositories and accession number(s) can be found: data and materials can be obtained at https://ukbiobank.dnanexus.com/panx/projects.
