# Supplementary material for: Association of sugary beverages consumption with liver fat content and fibro-inflammation: a large cohort study
Source: Front Public Health. 2025 Oct 9;13:1624848. doi: 10.3389/fpubh.2025.1624848 (PMC12545088; doi:10.3389/fpubh.2025.1624848)
Supplement: Supplementary file 1 [file Table_1.DOCX]

**Supplementary materials**

**1. Data assessment**

In the UK Biobank database, a wide range of covariates were collected and categorized.

(1) Beverage consumption was divided into sugar-sweetened beverages (SSB, including carbonated drinks and fruit drinks like J2O, squash, and cordial), artificially sweetened beverages (ASB, such as low-calorie or diet drinks), and natural juices (NJs, including pure orange juice, grapefruit juice, and other pure fruit/vegetable juices).

(2) Demographic and socioeconomic: age was evaluated by birthday and the date of recruitment. The Townsend Deprivation Index was based on the participants' residential postcode, incorporating the information on employment status, ownership of a car and home, and household crowding household income (<18,000; 18,000 to 30,999; 31,000 to 51,999; 52,000 to 100,000; >100,000 £/year) [1]. Education statues were divided into four classifications: college or university degree, A levels/AS levels or equivalent, O levels/GCSEs or equivalent, other.

(3) Lifestyle factors: physical activity was measured in metabolic equivalent (MET) hours per day to sum up all types of activities, including mild, moderate and vigorous activity. Hypertension was estimated by systolic blood pressure ≥140 mmHg or diastolic blood pressure ≥90 mmHg. BMI was calculated by dividing weight by height squared (kg/m^2^) and was categorized into normal weight, overweight and obesity (<25.0, 25.0 to <30, ≥30 kg/m^2^). Abdominal obesity was defined waist circumstance ≥102 cm for males and ≥88 cm for females [2].

(4) biochemical markers: glucose (mmol/L), triglyceride (mmol/L), cholesterol (mmol/L), C-reactive protein (mg/L) and platelet distribution width (%) were obtained from blood sample at recruitment with certain protocols.

(5) Dietary factors: total energy intake (kcal/d) and sugar intake (g/d) were assessed according to the food that participants consumed last 24 hours[3]. Healthy diet score were based on following criterion [4]: total vegetables, ≥ 4 servings per day; total fruit, ≥ 4 servings per day;total fish, ≥ 2 servings per week; processed meat, ≤ 1 servings per week; red meat, ≤ 1.5 servings per week; whole grains, ≥ 3 servings per day; refined grains, ≤ 1.5 servings per day and achieving one of the above criteria is scored as one point (ranged from 0-7).

(6) Oral health: periodontitis was assessed via touchscreen questionnaire, with participants reporting painful, bleeding, or loose gums being classified as having periodontitis [5].

**2. Linear regression analysis**

We performed univariate and multivariate linear regression models and calculated the beta coefficients and 95% confidence intervals (CIs). Since proton density fat fraction (PDFF) is skewed, we performed a logarithmic transformation on it before conducting linear regression. The transformed data approximates a normal distribution, meeting the assumptions required for linear regression analysis.

The specific steps are as follows: We used the logarithmically transformed PDFF (denoted as LN-PDFF) as the dependent variable and SBs consumption as the independent variable to establish a linear regression model. Through regression analysis, we calculated the regression coefficient β corresponding to LN-PDFF when SBs consumption increases by one unit. This indicates that when SBs consumption increases by one unit, the expected value of LN-PDFF will increase by β units.

To convert the results back to the actual change in PDFF, we need to perform a reverse transformation on the β value. Specifically, we exponentiated β (e^β^), which represents the multiple by which PDFF changes compared to the baseline when SBs consumption increases by one unit. For example, if β=0.1, then ^e0.1^=1.105, meaning that when SBs consumption increases by one unit, PDFF will increase by approximately 10.5%.

Finally, to calculate the arithmetic mean difference (AMD) in PDFF , we multiply the baseline PDFF value by e^β^ to obtain the new PDFF value, and then subtract the baseline PDFF value from it. The formula is as follows: AMD=(baseline PDFF×e^β^) − baseline PDFF.

Four linear regression models were conducted to investigate the association between beverages intake and PDFF: (1) model 1 was unadjusted; (2) model 2 was adjusted for age and sex; (3) model 3 was adjusted for age, sex, Townsend Deprivation Index, education, alcohol intake, smoking status, hypertension, physical activity, laboratory measurements (glucose, triglyceride, cholesterol, C-reactive protein and platelet distribution width) and dietary intake (total energy, total sugar, and healthy diet score). (4) Model 4 was fully adjusted for BMI and abdominal obesity based on Model 3.

**3. Quantile G-computation**

Quantile G-computation (QGC) model [6] is a robust statistical technique designed to estimate the cumulative impact of multiple exposure variables on health outcomes. It is a useful model to clarify the overall effect of the total beverage consumption pattern and the relative contribution of each type of beverage, rather than the effect of any single beverage in isolation. QCG directly yields an easily interpretable parameter—specifically, how the overall outcome changes for each one-unit increase in total beverage intake—along with the corresponding proportion of the effect attributable to each component. This facilitates policy-making and public health communication. In addition, QGC is especially advantageous when analyzing complex exposure data characterized by non-linear relationships or non-normal distributions. In our study, we utilized QGC to investigate the relationship between Proton Density Fat Fraction (PDFF) and beverage intake, modeled as continuous variables. We sequentially incorporated four beverage categories into the QGC model: artificially sweetened beverages (ASB), sugar-sweetened beverages (SSB), natural juices (NJs), and water. By doing so, we aimed to elucidate the joint association and relative significance of these beverages on PDFF. We assessed these relationships in both unadjusted and fully adjusted models to evaluate the influence of potential confounding factors.

**4. Substitution analysis**

In food substitution models, employing servings as the unit of substitution is a widely adopted practice. The core of this approach is to simulate the increase in the intake of one type of food while decreasing the intake of another type of food, in order to assess the impact of this substitution on health outcomes. Thus, we performed a substitution analysis to assess the impact of exchanging one type of beverage with another [7]. We assessed the relationship between swapping a serving of beverage for another by containing both as continuous variables in the multivariable linear regression model [8]. The difference in their beta coefficients indicated the effects of substitution. Utilizing the variances and the covariance matrix of these coefficients, we derived the 95% confidence intervals and the P value for the specific substitution effect estimate.

**5. Mediation analysis**

Mediation analysis were conducted to estimate how some factors mediated the relationship between ASB/SSB intake and PDFF, including BMI, sugar intake, abdominal obesity, healthy diet score, platelet distribution width, C-reactive protein, glucose, cholesterol, triglycerides and periodontitis. In the structural equations built by R software, there were three effects [9]: (1) total effect: the direct effect of the independent variable on the dependent variable, without considering the role of the mediator variable; (2) direct effect: the remaining direct effect of the independent variable on the dependent variable, after considering the mediator variable; (3) indirect effect: the indirect influence of the independent variable on the dependent variable through the mediator variable. The proportion of the mediating effect was calculated by dividing the regression coefficient of the indirect effect by the total effect coefficient.

**6. Reference**

[1] B. Jarman, P. Townsend, and V. Carstairs, Deprivation indices. Bmj 303 (1991) 523.

[2] M.E. Rinella, J.V. Lazarus, V. Ratziu, S.M. Francque, A.J. Sanyal, F. Kanwal, D. Romero, M.F. Abdelmalek, Q.M. Anstee, J.P. Arab, M. Arrese, R. Bataller, U. Beuers, J. Boursier, E. Bugianesi, C.D. Byrne, G.E. Castro Narro, A. Chowdhury, H. Cortez-Pinto, D.R. Cryer, K. Cusi, M. El-Kassas, S. Klein, W. Eskridge, J. Fan, S. Gawrieh, C.D. Guy, S.A. Harrison, S.U. Kim, B.G. Koot, M. Korenjak, K.V. Kowdley, F. Lacaille, R. Loomba, R. Mitchell-Thain, T.R. Morgan, E.E. Powell, M. Roden, M. Romero-Gómez, M. Silva, S.P. Singh, S.C. Sookoian, C.W. Spearman, D. Tiniakos, L. Valenti, M.B. Vos, V.W. Wong, S. Xanthakos, Y. Yilmaz, Z. Younossi, A. Hobbs, M. Villota-Rivas, and P.N. Newsome, A multisociety Delphi consensus statement on new fatty liver disease nomenclature. Hepatology 78 (2023) 1966-1986.

[3] A. Perez-Cornago, Z. Pollard, H. Young, M. van Uden, C. Andrews, C. Piernas, T.J. Key, A. Mulligan, and M. Lentjes, Description of the updated nutrition calculation of the Oxford WebQ questionnaire and comparison with the previous version among 207,144 participants in UK Biobank. Eur J Nutr 60 (2021) 4019-4030.

[4] W. Liu, T. Wang, M. Zhu, and G. Jin, Healthy Diet, Polygenic Risk Score, and Upper Gastrointestinal Cancer Risk: A Prospective Study from UK Biobank. Nutrients 15 (2023).

[5] P.I. Eke, B.A. Dye, L. Wei, G.D. Slade, G.O. Thornton-Evans, J.D. Beck, G.W. Taylor, W.S. Borgnakke, R.C. Page, and R.J. Genco, Self-reported measures for surveillance of periodontitis. J Dent Res 92 (2013) 1041-7.

[6] A.P. Keil, J.P. Buckley, K.M. O'Brien, K.K. Ferguson, S. Zhao, and A.J. White, A Quantile-Based g-Computation Approach to Addressing the Effects of Exposure Mixtures. Environ Health Perspect 128 (2020) 47004.

[7] D.B. Ibsen, A.S.D. Laursen, A.M.L. Würtz, C.C. Dahm, E.B. Rimm, E.T. Parner, K. Overvad, and M.U. Jakobsen, Food substitution models for nutritional epidemiology. Am J Clin Nutr 113 (2021) 294-303.

[8] L. Ma, Y. Hu, D.J. Alperet, G. Liu, V. Malik, J.E. Manson, E.B. Rimm, F.B. Hu, and Q. Sun, Beverage consumption and mortality among adults with type 2 diabetes: prospective cohort study. Bmj 381 (2023) e073406.

[9] C.M. Stein, N.J. Morris, N.B. Hall, and N.L. Nock, Structural Equation Modeling. Methods Mol Biol 1666 (2017) 557-580.

Table S1. Definitions and codes used for defining existing liver disease at baseline.

| **Liver Disease at Baseline** | **Description** | **ICD10** | **Data Field** | **Assessment Centre Data** |
| --- | --- | --- | --- | --- |
| **Liver Disease** | Any alcoholic liver disease | K70 | 41270 | International Classification of Diseases and related health problems |
|  | Any toxic liver disease | K71 |  |  |
|  | Hepatic failure, not elsewhere classified | K72 |  |  |
|  | Chronic hepatitis, not elsewhere classified | K73 |  |  |
|  | Fibrosis and cirrhosis of liver | K74 |  |  |
|  | Other inflammatory liver diseases | K75 |  |  |
|  | Other diseases of liver | K76 |  |  |
|  | Liver failure/ cirrhosis | / | 20002 | Verbal interview |
|  | Infective/viral hepatitis | / |  |  |
|  | Non-infective hepatitis | / |  |  |
|  | Hepatitis | / |  |  |

Table S2. Baseline characteristics of participants by artificially-sweetened beverages and nature juices beverages intake.

| **Characteristics** | **Artificially-sweetened Beverages** | | | **P value** | **Nature Juices** | | | **P value** |
| --- | --- | --- | --- | --- | --- | --- | --- | --- |
|  | **0/d** | **0-1/d** | **≥1/d** |  | **0/d** | **0-1/d** | **≥1/d** |  |
| **Sample size, n(%)** | 20374 (78.7%) | 664 (2.5%) | 4820 (18.6%) |  | 11318 (43.7%) | 6021 (23.2%) | 8519 (32.9%) |  |
| **Male, n(%)** | 9865 (48.4%) | 303 (45.6%) | 2026 (42.7%) | <0.001 | 5028 (44.4%) | 2992 (49.7%) | 4208 (49.4%) | <0.001 |
| **Age (years)** | 55.82 (7.45) | 55.31 (7.36) | 53.24 (7.46) | <0.001 | 54.86 (7.45) | 56.53 (7.36) | 54.72 (7.61) | <0.001 |
| **Townsend deprivation Index** | -1.89 (2.72) | -1.96 (2.71) | -1.98 (2.65) | 0.524 | -1.80 (2.77) | -2.15 (2.56) | -1.85 (2.73) | <0.001 |
| **Education** |  |  |  | <0.001 |  |  |  | <0.001 |
| College or  University degree | 10549 (51.8%) | 320 (48.2%) | 2026 (47.3%) |  | 5084 (44.9%) | 3216 (53.4%) | 4850 (56.9%) |  |
| A AS level or equivalent | 2654 (13.0%) | 95 (14.3%) | 6026 (14.2%) |  | 1551 (13.7%) | 806 (13.4%) | 1077 (12.6%) |  |
| O levels or equivalent | 4218 (20.7%) | 144 (21.7%) | 1026 (23.8%) |  | 2817 (24.9%) | 1161 (19.3%) | 1533 (18.0%) |  |
| Other | 2953 (14.5%) | 105 (15.8%) | 7026 (14.6%) |  | 1866 (16.5%) | 838 (13.9%) | 1059 (12.4%) |  |
| **Alcohol intake (g/d)** | 10.96 (9.75) | 11.12 (10.5) | 10.83 (9.59) | 0.526 | 10.86 (9.83) | 10.97 (9.41) | 11.01 (9.89) | 0.182 |
| **Smoking status,n(%)** |  |  |  | 0.436 |  |  |  | <0.001 |
| Never | 12529 (61.5%) | 391 (58.9%) | 2026 (60.5%) |  | 6596 (58.3%) | 3783 (62.8%) | 5459 (64.1%) |  |
| Former | 1176 (5.8%) | 47 (7.1%) | 2026 (6.1%) |  | 766 (6.8%) | 283 (4.7%) | 466 (5.5%) |  |
| Current | 6669 (32.7%) | 226 (34.0%) | 1026 (33.4%) |  | 3956 (35.0%) | 1955 (32.5%) | 2594 (30.4%) |  |
| **Abdominal obesity, n(%)** | 4319 (21.2%) | 200 (30.1%) | 1026 (33.1%) | <0.001 | 2910 (25.7%) | 1267 (21.0%) | 1936 (22.7%) | <0.001 |
| **BMI (kg/m^2^ )** | 25.86 (3.85) | 26.75 (4.31) | 27.50 (4.48) | <0.001 | 26.47 (4.23) | 25.97 (3.85) | 26.57 (4.02) | <0.001 |
| **Physical activity (MET hours/week)** | 40.62 (36.53) | 40.65 (37.1) | 39.48 (36.29) | <0.001 | 40.59 (37.27) | 40.17 (35.65) | 40.32 (36.30) | 0.244 |
| **Hypertension, n(%)** | 7991 (39.2%) | 261 (39.3%) | 1026 (36.7%) | 0.005 | 4257 (37.6%) | 2401 (39.9%) | 3363 (39.5%) | 0.004 |
| **Diabetes, n(%)** | 438 (2.1%) | 30 (4.5%) | 2026 (4.3%) | <0.001 | 368 (3.3%) | 147 (2.4%) | 161 (1.9%) | <0.001 |
| **Albumin (g/L)** | 45.49 (2.52) | 45.37 (2.39) | 45.40 (2.55) | <0.001 | 45.46 (2.51) | 45.40 (2.52) | 45.52 (2.53) | <0.001 |
| **Glucose (mmol/L)** | 5.00 (0.87) | 5.12 (1.30) | 5.02 (1.15) | <0.001 | 5.02 (1.09) | 5.02 (0.89) | 5.05 (0.83) | <0.001 |
| **Triglyceride**  **(mmol/L)** | 1.60 (0.92) | 1.65 (1.02) | 1.64 (0.98) | <0.001 | 1.61 (0.96) | 1.59 (0.90) | 1.63 (0.93) | <0.001 |
| **Cholesterol (mmol/L)** | 5.76 (1.09) | 5.63 (1.15) | 5.84 (1.11) | <0.001 | 5.71 (1.10) | 5.75 (1.09) | 5.72 (1.09) | <0.001 |
| **C-reactive protein (mg/L)** | 1.89 (3.45) | 1.99 (2.69) | 2.30 (3.84) | <0.001 | 2.02 (3.42) | 1.94 (3.42) | 1.96 (3.69) | <0.001 |
| **Platelet Count (10^9/^L)** | 248.9 (64.81) | 248.0 (64.32) | 251.7 (64.76) | <0.001 | 251.7 (65.33) | 246.2 (64.15) | 248.6 (64.56) | <0.001 |
| **HDL-c (mmol/L)** | 1.50 (0.38) | 1.47 (0.38) | 1.45 (0.38) | <0.001 | 1.49 (0.38) | 1.51 (0.38) | 1.48 (0.38) | <0.001 |
| **LDL-c (mmol/L)** | 3.59 (0.83) | 3.51 (0.86) | 3.53 (0.84) | <0.001 | 3.56 (0.84) | 3.59 (0.83) | 3.58 (0.83) | <0.001 |
| **ALT (U/L)** | 22.36 (13.22) | 22.55 (12.7) | 23.11 (13.80) | <0.001 | 22.45 (13.40) | 22.08 (12.62) | 22.93 (13.76) | <0.001 |
| **AST (U/L)** | 25.54 (9.09) | 25.65 (9.08) | 25.69 (8.86) | <0.001 | 25.55 (9.18) | 25.45 (9.36) | 25.71 (8.63) | <0.001 |
| **GGT (U/L)** | 32.15 (31.92) | 35.23 (32.3) | 32.24 (28.88) | <0.001 | 31.52 (26.95) | 32.47 (33.28) | 32.90 (34.03) | <0.001 |
| **Energy (kJ/d)** | 8729.66 (2039.72) | 8585.49 (2124) | 8806.65 (2066.21) | <0.001 | 8383.35 (2075.99) | 8752.08 (1941.69) | 8998.41 (2049.44) | <0.001 |
| **Sugar intake (g/d)** | 124.90 (40.56) | 123.82 (40.6) | 123.56 (41.59) | <0.001 | 112.79 (39.35) | 126.11 (36.78) | 136.07 (41.69) | <0.001 |
| **Healthy diet score** | 3.10 (1.36) | 3.00 (1.31) | 3.02 (1.36) | <0.001 | 3.04 (1.36) | 3.04 (1.34) | 3.15 (1.36) | <0.001 |

Abbreviations: BMI, body mass index; HDL-c, high-density lipoprotein cholesterol; LDL-c, low-density lipoprotein cholesterol; ALT, alkaline phosphatase; AST,glutamic oxaloacetic transaminase; GGT, glutamyl transpeptidase; MET, metabolic equivalent.

Table S3. Linear regression models were performed to analyze the association between category of beverages intake and PDFF.

| **Category of Beverage intake** | **Model 1** | | **Model 2** | | **Model 3** | | **Model 4** | |
| --- | --- | --- | --- | --- | --- | --- | --- | --- |
|  | **Difference (95%Cl)** | **P** | **Difference (95%Cl)** | **P** | **Difference (95%Cl)** | **P** | **Difference (95%Cl)** | **P** |
| **Artificially-sweetened Beverage** | |  |  |  |  |  |  |  |
| 0 serving/d | reference |  | reference |  | reference |  | reference |  |
| 0-1 serving/d | 0.36 (0.10, 0.63) | 0.006 | 0.41 (0.15, 0.67) | <0.001 | 0.19 (-0.03, 0.43) | 0.096 | -0.04 (-0.24, 0.17) | 0.696 |
| ≥1 serving/d | 0.62 (0.51, 0.74) | <0.001 | 0.76 (0.65, 0.88) | <0.001 | 0.55 (0.45, 0.66) | <0.001 | 0.15 (0.06, 0.24) | <0.001 |
| Per 1 serving/d increased | 0.33 (0.27, 0.39) | <0.001 | 0.39 (0.33, 0.45) | <0.001 | 0.25 (0.20, 0.30) | <0.001 | 0.07 (0.02, 0.12) | 0.003 |
| **Sugar-sweetened Beverages** | |  |  |  |  |  |  |  |
| 0 serving/d | reference |  | reference |  | reference |  | reference |  |
| 0-1 serving/d | 0.18 (0.01, 0.35) | 0.037 | 0.15 (-0.01, 0.31) | <0.072 | 0.17 (0.02, 0.32) | 0.028 | 0.19 (0.05, 0.34) | 0.007 |
| ≥1 serving/d | 0.38 (0.29, 0.48) | <0.001 | 0.32 (0.23, 0.42) | <0.001 | 0.30 (0.21, 0.39) | <0.001 | 0.21 (0.12, 0.29) | <0.001 |
| Per 1 serving/d increased | 0.26 (0.20, 0.31) | <0.001 | 0.21 (0.15, 0.26) | <0.001 | 0.18 (0.13, 0.23) | <0.001 | 0.12 (0.07, 0.16) | <0.001 |
| **Nature Juices** | |  |  |  |  |  |  |  |
| 0 serving/d | reference |  | reference |  | reference |  | reference |  |
| 0-1 serving/d | -0.30 (-0.40, -0.19) | <0.001 | -0.39 (-0.48, -0.29) | <0.001 | -0.23 (-0.33, -0.14) | <0.001 | -0.10 (-0.19, -0.01) | 0.027 |
| ≥1 serving/d | -0.14 (-0.23, -0.04) | 0.007 | -0.21 (-0.30, -0.12) | <0.001 | -0.05 (-0.14, 0.04) | 0.291 | 0.01 (-0.07, 0.10) | 0.706 |
| Per 1 serving/d increased | -0.05 (-0.13, 0.02) | 0.150 | -0.13 (-0.19, -0.06) | <0.001 | 0.00 (-0.06, 0.07) | 0.952 | 0.03 (-0.03, 0.09) | 0.348 |

Model 1: Unadjusted.

Model 2: Adjusted for age and sex.

Model 3: Model 2 plus deprivation Index, education, alcohol intake, smoking status, hypertension, physical activity, laboratory measurements (glucose, triglyceride, cholesterol, C-reactive protein and platelet distribution width), dietary intake (total energy, total sugar, and healthy diet score).

Model 4: Model 3 plus body mass index and abdominal obesity.

Table S4. Linear regression models were performed to analyze the association between category of beverages intake and CT1.

| **Category of Beverage intake** | **Model 1** | | **Model 2** | | **Model 3** | | **Model 4** | |
| --- | --- | --- | --- | --- | --- | --- | --- | --- |
|  | **Difference (95%Cl)** | **P** | **Difference (95%Cl)** | **P** | **Difference (95%Cl)** | **P** | **Difference (95%Cl)** | **P** |
| **Artificially-sweetened Beverage** | |  |  |  |  |  |  |  |
| 0 serving/d | reference |  | reference |  | reference |  | reference |  |
| 0-1 serving/d | 8.48 (6.63, 10.34) | <0.001 | 9.95 (8.09, 11.80) | <0.001 | 7.46 (5.69, 9.25) | <0.001 | 2.48 (0.75, 4.21) | 0.005 |
| ≥1 serving/d | 9.32 (4.82, 13.82) | <0.001 | 9.99 (5.54, 14.44) | <0.001 | 7.06 (2.79, 11.34) | <0.001 | 3.86 (1.26, 6.79) | <0.001 |
| Per 1 serving/d increased | 5.06 (4.08, 6.05) | <0.001 | 5.75 (4.76, 6.73) | <0.001 | 4.37 (3.42, 5.32) | <0.001 | 1.67 (0.76, 2.59) | <0.001 |
| **Sugar-sweetened Beverages** | |  |  |  |  |  |  |  |
| 0 serving/d | reference |  |  |  |  |  | reference |  |
| 0-1 serving/d | 5.80 (2.83, 8.77) | <0.001 | 5.87 (4.25, 7.50) | <0.001 | 3.32 (1.70, 4.93) | <0.001 | 1.94 (0.40, 3.49) | 0.014 |
| ≥1 serving/d | 6.34 (4.70, 7.97) | <0.001 | 5.42 (2.47, 8.34) | <0.001 | 4.35 (1.51, 7.19) | <0.001 | 2.43 (1.31, 3.57) | <0.001 |
| Per 1 serving/d increased | 3.59 (3.01, 4.90) | <0.001 | 3.58 (2.64, 4.52) | <0.001 | 1.97 (1.03, 2.90) | <0.001 | 1.06 (0.35, 1.97) | 0.007 |
| **Nature Juices** | |  |  |  |  |  |  |  |
| 0 serving/d | reference |  | reference |  | reference |  | reference |  |
| 0-1 serving/d | -2.03 (-3.88, -0.19) | 0.031 | -3.14 (-4.98, -1.30) | <0.001 | -1.72 (-3.50, 0.06) | 0.059 | -0.14 (-1.86, 1.56) | 0.866 |
| ≥1 serving/d | 0.35 (-1.34, 2.04) | 0.684 | -0.36 (-2.04, 1.31) | 0.671 | -0.07 (-1.73, 1.60) | 0.928 | 0.77 (-0.84, 2.38) | 0.394 |
| Per 1 serving/d increased | 1.27 (0.02, 2.57) | 0.045 | 0.57 (-0.66, 1.79) | 0.364 | 0.571 (-0.66, 1.80) | 0.364 | 0.94 (-0.23, 2.12) | 0.116 |

Model 1: Unadjusted.

Model 2: Adjusted for age and sex.

Model 3: Model 2 plus deprivation Index, education, alcohol intake, smoking status, hypertension, physical activity, laboratory measurements (glucose, triglyceride, cholesterol, C-reactive protein and platelet distribution width), dietary intake (total energy, total sugar, and healthy diet score).

Model 4: Model 3 plus body mass index and abdominal obesity.

Table S5. Logistic regression models were performed to analyze the association between category of beverages intake and threshold of PDFF and cT1.

| **Category of Beverage intake** | **PDFF** | | | | **cT1** | | | |
| --- | --- | --- | --- | --- | --- | --- | --- | --- |
|  | **Unadjusted**  **Difference (95%Cl)** | **P** | **Adjusted**  **Difference (95%Cl)** | **P** | **Unadjusted**  **Difference (95%Cl)** | **P** | **Adjusted**  **Difference (95%Cl)** | **P** |
| **Artificially-sweetened Beverage** | |  |  |  |  |  |  |  |
| 0 serving/d | reference |  | reference |  | reference |  | reference |  |
| 0-1 serving/d | 1.22 (1.02, 1.44) | 0.024 | 0.96 (0.79, 1.16) | 0.702 | 1.64 (1.42, 1.89) | <0.001 | 1.16 (1.00, 1.35) | 0.048 |
| ≥1 serving/d | 1.37 (1.28, 1.47) | <0.001 | 1.08 (1.03, 1.15) | 0.005 | 1.74 (1.24, 2.38) | <0.001 | 1.33 (1.10, 1.56) | <0.001 |
| Per 1 serving/d increased | 1.16 (1.10, 1.29) | <0.001 | 1.04 (1.01, 1.08) | <0.001 | 1.34 (1.13, 1.62) | <0.001 | 1.13 (1.03, 1.22) | <0.001 |
| **Sugar-sweetened Beverages** | |  |  |  |  |  |  |  |
| 0 serving/d | reference |  | reference |  | reference |  | reference |  |
| 0-1 serving/d | 1.11 (0.99, 1.24) | 0.076 | 1.13 (0.99, 1.28) | 0.063 | 1.20 (0.92, 1.53) | 0.201 | 1.18 (0.90, 1.53) | 0.354 |
| ≥1 serving/d | 1.21 (1.14, 1.29) | <0.001 | 1.14 (1.02, 1.23) | 0.008 | 1.51 (1.31, 1.71) | <0.001 | 1.29 (1.12, 1.48) | <0.001 |
| Per 1 serving/d increased | 1.10 (1.03, 1.17) | <0.001 | 1.05 (1.01, 1.10) | 0.002 | 1.25 (1.15, 1.32) | <0.001 | 1.14 (1.06, 1.26) | <0.001 |
| **Nature Juices** | |  |  |  |  |  |  |  |
| 0 serving/d | reference |  | reference |  | reference |  | reference |  |
| 0-1 serving/d | 0.82 (0.77, 0.88) | <0.001 | 0.91 (0.84, 0.98) | 0.019 | 0.83 (0.71, 0.98) | 0.027 | 1.02 (0.86, 1.21) | 0.834 |
| ≥1 serving/d | 0.91 (0.85, 0.96) | 0.002 | 0.97 (0.90, 1.04) | 0.447 | 0.98 (0.85, 1.13) | 0.893 | 1.15 (0.99, 1.33) | 0.073 |
| Per 1 serving/d increased | 0.94 (0.90, 0.97) | 0.128 | 0.99 (0.92, 1.05) | 0.639 | 0.95 (0.87, 1.05) | 0.359 | 1.07 (0.95, 1.20) | 0.890 |

Models were adjusted for age, sex, Deprivation Index, education, alcohol intake, smoking status, hypertension, diabetes, physical activity, laboratory measurements (glucose, triglyceride, cholesterol, C-reactive protein and platelet distribution width), dietary intake (total energy, total sugar, and healthy diet score), body mass index and abdominal obesity.

Table S6. Age subgroup analyses of association between category of beverages intake with PDFF and CT1.

| **Category of Beverages intake** | **PDFF** | |  | **CT1** | |  |
| --- | --- | --- | --- | --- | --- | --- |
|  | **Age <55** | **Age ≥55** | **P for interaction** | **Age <55** | **Age ≥55** | **P for interaction** |
|  | **Difference (95%Cl)** | **Difference (95%Cl)** |  | **Difference (95%Cl)** | **Difference (95%Cl)** |  |
| **Artificially-sweetened Beverages** | |  |  |  |  |  |
| 0 serving/d | reference | reference |  | reference | reference |  |
| 0-1 serving/d | 0.06 (-0.10, 0.23) | 0.13 (-0.02, 0.29) | 0.113 | 8.16 (1.69, 14.63) | 0.59 (-4.70, 5.89) | 0.061 |
| ≥1 serving/d | 0.13 (-0.05, 0.32) | 0.18 (0.05, 0.32) |  | 2.09 (0.65, 5.54) | 1.23 (-1.20, 3.67) |  |
| **Sugar-sweetened Beverages** | |  |  |  |  |  |
| 0 serving/d | reference | reference | 0.322 |  |  |  |
| 0-1 serving/d | 0.08 (-0.07, 0.22) | 0.25 (0.12, 0.38) |  | 1.82 (-2.53, 6.18) | 6.41 (2.59, 9.88) | 0.596 |
| ≥1 serving/d | 0.16 (0.05, 0.26) | 0.27 (0.15, 0.38) |  | 2.36 (0.04, 4.69) | 1.54 (-0.51, 3.60) |  |
| **Nature juices** | |  |  |  |  |  |
| 0 serving/d | reference | reference |  |  |  |  |
| 0-1 serving/d | -0.07 (-0.21, 0.06) | 0.03 (-0.08, 0.15) | 0.842 | -1.64 (-4.39, 1.11) | 0.97 (-1.18, 3.13) | 0.378 |
| ≥1 serving/d | -0.02 (-0.26, 0.22) | -0.11 (-0.32, 0.10) |  | 0.95 (-1.48, 3.39) | 0.88 (-1.24, 3.01) |  |

Table S7. Sex subgroup analyses of association between category of beverages intake with PDFF and CT1.

| **Category of Beverages intake** | **PDFF** | |  | **CT1** | |  |
| --- | --- | --- | --- | --- | --- | --- |
|  | **Male** | **Female** | **P for interaction** | **Male** | **Female** | **P for interaction** |
|  | **Difference (95%Cl)** | **Difference (95%Cl)** |  | **Difference (95%Cl)** | **Difference (95%Cl)** |  |
| **Artificially-sweetened Beverages** | |  |  |  |  |  |
| 0 serving/d | reference | reference |  | reference | reference |  |
| 0-1 serving/d | 0.22 (0.02, 0.44) | 0.04 (-0.08, 0.16) | 0.513 | 3.05 (0.37, 5.74) | 1.41 (-3.92, 6.76) | 0.517 |
| ≥1 serving/d | 0.32 (0.07, 0.58) | 0.13 (-0.02, 0.28) |  | 7.22 (0.85, 13.59) | 2.21 (-0.02, 4.45) |  |
| **Sugar-sweetened Beverages** | |  |  |  |  |  |
| 0 serving/d | reference | reference |  | reference | reference |  |
| 0-1 serving/d | 0.21 (0.05, 0.38) | 0.13 (0.03, 0.24) | 0.757 | 2.73 (0.44, 5.02) | 1.04 (-1.04, 3.13) | 0.552 |
| ≥1 serving/d | 0.31 (0.10, 0.53) | 0.31 (0.15, 0.48) |  | 5.63 (1.54, 9.71) | 3.11 (-0.53, 6.77) |  |
| **Nature juices** | |  |  |  |  |  |
| 0 serving/d | reference | reference |  | reference | reference |  |
| 0-1 serving/d | -0.10 (-0.25, 0.06) | 0.03 (-0.08, 0.15) | 0.465 | -0.02 (-3.30, 1.94) | 0.10 (-2.18, 2.39) | 0.734 |
| ≥1 serving/d | -0.18 (-0.43, 0.09) | -0.11 (-0.32, 0.10) |  | 0.04 (-2.37, 2.46) | 1.38 (-0.77, 3.54) |  |

Table S8. BMI subgroup analyses of association between category of beverages intake with PDFF and CT1.

| **Category of Beverages intake** |  | **PDFF** |  |  |  | **CT1** |  |  |
| --- | --- | --- | --- | --- | --- | --- | --- | --- |
|  | **Normal weight** | **Overweight** | **Obesity** | **P for interaction** | **Normal weight** | **Overweight** | **Obesity** | **P for interaction** |
|  | **Difference (95%Cl)** | **Difference (95%Cl)** | **Difference (95%Cl)** |  | **Difference (95%Cl)** | **Difference (95%Cl)** | **Difference (95%Cl)** |  |
| **Artificially-sweetened Beverages** | |  |  |  |  |  |  |  |
| 0 serving/d | reference | reference | reference |  | reference | reference | reference |  |
| 0-1 serving/d | -0.09 (-0.28, 0.10) | -0.20 (-0.55, 0.16) | 0.82 (-0.19, 1.97) | 0.699 | 0.94 (-1.67, 3.56) | 2.40 (-0.21, 5.02) | 4.39 (-0.16, 8.59) | 0.001 |
| ≥1 serving/d | 0.10 (0.02, 0.19) | 0.17 (0.01, 0.33) | 0.08 (-0.31, 0.49) |  | -5.36 (-11.47, 0.09） | 7.87 (1.67, 14.07) | 10.91 (-0.08, 21.91) |  |
| **Sugar-sweetened Beverages** | |  |  |  |  |  |  |  |
| 0 serving/d | reference | reference | reference |  | reference | reference | reference |  |
| 0-1 serving/d | 0.10 (0.00, 0.22) | 0.30 (0.04, 0.58) | 0.11 (-0.60, 0.89) | 0.261 | 3.27 (-0.40, 6.95) | 6.89 (2.56, 11.22) | 0.97 (-7.45, 9.40) | <0.001 |
| ≥1 serving/d | 0.12 (0.05, 0.19) | 0.21 (0.07, 0.36) | 0.43 (0.02, 0.86) |  | -0.50 (-2.63, 1.62) | 2.88 (0.48, 5.28) | 5.71 (1.08, 10.34) |  |
| **Nature juices** | |  |  |  |  |  |  |  |
| 0 serving/d | reference | reference | reference |  | reference | reference | reference |  |
| 0-1 serving/d | -0.03 (-0.10, 0.04) | -0.16 (-0.31, -0.01) | -0.19 (-0.65, 0.30) | 0.108 | -0.66 (-2.94, 1.60) | 0.10 (-2.58, 2.80) | 1.15 (-4.23, 6.35) | 0.198 |
| ≥1 serving/d | 0.05 (-0.02, 0.12) | -0.07 (-0.21, 0.08) | 0.11 (-0.33, 0.55) |  | 0.82 (-1.36, 3.01) | 0.06 (-2.44, 2.57) | 3.36 (-1.55, 8.27) |  |

Table S9. Physical activity subgroup analyses of association between category of beverages intake with PDFF and CT1.

| **Category of Beverages intake** | **PDFF** | |  | **cT1** | |  |
| --- | --- | --- | --- | --- | --- | --- |
|  | **Under guideline recommendations** | **Meeting guideline recommendations** | **P for interaction** | **Under guideline recommendations** | **Meeting guideline recommendations** | **P for interaction** |
|  | **Difference (95%Cl)** | **Difference (95%Cl)** |  | **Difference (95%Cl)** | **Difference (95%Cl)** |  |
| **Artificially-sweetened Beverages** | |  |  |  |  |  |
| 0 serving/d | reference | reference |  | reference | reference |  |
| 0-1 serving/d | -0.07 (-0.40, 0.29) | -0.03 (-0.27, 0.24) | 0.629 | 2.81 (0.09, 5.54) | 2.22 (0.01, 4.45) | 0.349 |
| ≥1 serving/d | 0.17 (0.01, 0.32) | 0.13 (0.03, 0.25) |  | 1.55 (-4.83, 7.94) | 5.66 (0.28, 11.04) |  |
| **Sugar-sweetened Beverages** | |  |  |  |  |  |
| 0 serving/d | reference | reference |  | reference | reference |  |
| 0-1 serving/d | 0.21 (0.05, 0.38) | 0.17 (0.00, 0.34) | 0.301 | 3.18 (0.74, 5.63) | 0.98 (-1.00, 2.98) | 0.122 |
| ≥1 serving/d | 0.31 (0.10, 0.53) | 0.17 (0.07, 0.27) |  | 4.55 (0.27, 8.83) | 4.21 (0.75, 7.83) |  |
| **Nature juices** | |  |  |  |  |  |
| 0 serving/d | reference | reference |  | reference | reference |  |
| 0-1 serving/d | -0.01 (-0.05, 0.06) | 0.03 (-0.08, 0.15) | 0.249 | 1.36 (-1.35,4.07) | -1.26 (-3.46, 0.93) | 0.526 |
| ≥1 serving/d | -0.15 (-0.40, 0.09) | -0.01 (-0.12, 0.10) |  | 1.25 (-1.28, 3.80) | 0.46 (-1.61, 2.54) |  |

Meeting guideline recommendations indicates exceeded 150 minutes of moderate activity per week or 75 minutes of vigorous activity

Table S10. Alcohol consumption subgroup analyses of association between category of beverages intake with PDFF and cT1.

| **Category of Beverages intake** | **PDFF** | |  | **cT1** | |  |
| --- | --- | --- | --- | --- | --- | --- |
|  | **<10g/d** | **≥10g/d** | **P for interaction** | **<10g/d** | **≥10g/d** | **P for interaction** |
|  | **Difference (95%Cl)** | **Difference (95%Cl)** |  | **Difference (95%Cl)** | **Difference (95%Cl)** |  |
| **Artificially-sweetened Beverages** | |  |  |  |  |  |
| 0 serving/d | reference | reference |  | reference | reference |  |
| 0-1 serving/d | -0.05 (-0.31, 0.22) | -0.02 (-0.33, 0.31) | 0.002 | 3.47 (-1.93, 8.87) | 3.91 (-2.43, 10.26) | 0.113 |
| ≥1 serving/d | 0.05 (-0.08, 0.18） | 0.19 (0.08, 0.32） |  | 2.20 (-0.08, 4.50) | 2.96 (0.34, 5.59) |  |
| **Sugar-sweetened Beverages** | |  |  |  |  |  |
| 0 serving/d | reference | reference |  | reference | reference |  |
| 0-1 serving/d | 0.04 (-0.17, 0.25） | 0.29 (0.11, 0.48） | <0.001 | 5.15 (1.59, 8.70) | 3.67 (-0.56, 7.11) | 0.409 |
| ≥1 serving/d | 0.16 (0.04, 0.28） | 0.24 (0.14, 0.35） |  | 2.10 (0.07, 4.12) | 1.60 (-0.79, 4.00) |  |
| **Nature juices** | |  |  |  |  |  |
| 0 serving/d | reference | reference |  | reference | reference |  |
| 0-1 serving/d | -0.12 (-0.22, 0.00) | -0.05 (-0.16, 0.07) | 0.435 | 0.20 (-2.04, 2.45) | -0.58 (-3.22, 2.05) | 0.651 |
| ≥1 serving/d | -0.04 (-0.17, 0.09) | 0.09 (-0.02, 0.20) |  | 1.24 (-0.87, 3.36) | 0.23 (-2.24, 2.71) |  |

Table S11 . Moderating effect of confounding factors for the association between category of Beverages intake and PDFF.

| **Adjusted factor** | **Artificially-sweetened Beverages** | | | **Sugar-sweetened Beverages** | | |
| --- | --- | --- | --- | --- | --- | --- |
|  | **β** | **95% Cl** | **P for interaction** | **β** | **95% Cl** | **P for interaction** |
| Sex | 0.000 | (-0.001, 0.002) | 0.513 | 0.003 | (-0.016, 0.022) | 0.757 |
| Age | -0.017 | (-0.039, 0.004) | 0.113 | 0.001 | (-0.001, 0.002) | 0.322 |
| Deprivation Index | 0.001 | (-0.003, 0.005) | 0.511 | 0.005 | (0.002, 0.009) | 0.003* |
| Education | -0.006 | (-0.014, 0.001) | 0.105 | 0.006 | (-0.001, 0.013) | 0.075 |
| Alcohol intake (g/d) | 0.002 | (0.001, 0.003) | 0.002* | 0.002 | (0.001, 0.003) | <0.001* |
| Smoking status | -0.010 | (-0.021, 0.002) | 0.089 | 0.003 | (-0.007, 0.013) | 0.580 |
| Abdominal obesity | 0.009 | (-0.015, 0.032) | 0.473 | 0.031 | (0.009, 0.053) | 0.007* |
| BMI (kg/m^2^ ) | -0.003 | (-0.018, 0.012) | 0.699 | 0.008 | (-0.006, 0.021) | 0.261 |
| Physical activity (MET hours/week) | -0.008 | (-0.042, 0.025) | 0.629 | 0.000 | (-0.000,0.000) | 0.301 |
| Hypertension | 0.000 | (-0.022, 0.022) | 0.990 | 0.006 | (-0.014, 0.025) | 0.560 |
| C-reactive protein (mg/L) | -0.001 | (-0.003, 0.001) | 0.251 | 0.002 | (-0.000,0.004) | 0.078 |
| Platelet distribution width (%) | 0.000 | (-0.000,0.001) | 0.191 | -0.000 | (-0.000, 0.000) | 0.642 |
| Glucose (mmol/L) | 0.002 | (-0.008, 0.012) | 0.703 | 0.008 | (-0.002, 0.018) | 0.128 |
| Triglyceride (mmol/L) | -0.009 | (-0.020, 0.124) | 0.124 | 0.003 | (-0.001, 0.010) | 0.540 |
| Cholesterol (mmol/L) | -0.009 | (-0.020, 0.002) | 0.264 | 0.001 | (-0.007，0.010) | 0.721 |
| Energy (kJ/d) | 0.000 | (-0.001, 0.000) | 0.412 | 0.000 | (-0.001, 0.000) | 0.851 |
| Sugar intake (g/d) | 0.001 | (0.000, 0.001) | 0.009* | 0.001 | (0.000, 0.001) | 0.004* |
| healthy diet score | -0.005 | (-0.015, 0.005) | 0.322 | 0.004 | (-0.002, 0.011) | 0.208 |

Model was adjusted for age, sex, deprivation Index, education, alcohol intake, smoking status, hypertension, physical activity, laboratory measurements (glucose, triglyceride, cholesterol, C-reactive protein and platelet distribution width), dietary intake (total energy, total sugar, and healthy diet score) body mass index and abdominal obesity.

Table S12 . Moderating effect of confounding factors for the association between category of Beverages intake and cT1.

| **Adjusted factor** | **Artificially-sweetened Beverages** | | | **Sugar-sweetened Beverages** | | |
| --- | --- | --- | --- | --- | --- | --- |
|  | **β** | **95% Cl** | **P for interaction** | **β** | **95% Cl** | **P for interaction** |
| Sex | -0.55 | (-2.25, 1.13) | 0.517 | -0.04 | (-0.12, 0.06) | 0.552 |
| Age | -0.10 | (-0.22, 0.01) | 0.061 | -0.03 | (-1.88, 1.08) | 0.596 |
| Deprivation Index | 0.11 | (-0.20, 0.42) | 0.480 | 0.32 | (0.05, 0.59) | 0.020* |
| Education | -0.58 | (-1.32, 0.15) | 0.121 | 0.15 | (-0.49, 0.80) | 0.640 |
| Alcohol intake (g/d) | 0.06 | (-0.01, 0.14) | 0.113 | -0.03 | (-0.10, 0.04) | 0.409 |
| Smoking status | -0.65 | (-1.55, 0.23) | 0.150 | 0.317 | (-0.48, 1.11) | 0.436 |
| Abdominal obesity | 3.01 | (1.15, 4.86) | 0.001* | 4.37 | (2.56, 6.10) | <0.001* |
| BMI (kg/m^2^ ) | 1.59 | (0.80, 3.09) | 0.001* | 12.64 | (10.37, 14.55) | <0.001* |
| Physical activity (MET hours/week) | -0.01 | (-0.03, 0.01) | 0.349 | -0.01 | (-0.03, 0.01) | 0.122 |
| Hypertension | 0.48 | (-2.34, 3.11) | 0.726 | 0.49 | (-1.03, 2.01) | 0.510 |
| C-reactive protein (mg/L) | -0.09 | (-0.32, 0.13) | 0.405 | 0.18 | (-0.03, 0.39) | 0.100 |
| Platelet distribution width (%) | -0.01 | (-1.01, 1.59) | 0.987 | -0.74 | (-2.16, 0.67) | 0.306 |
| Glucose (mmol/L) | 0.89 | (0.12, 1.67) | 0.022* | 0.62 | (-2.38, 0.45) | 0.181 |
| Triglyceride (mmol/L) | 0.84 | (-0.03, 1.71) | 0.059 | 0.89 | (0.12, 1.66) | 0.023* |
| Cholesterol (mmol/L) | 0.81 | (0.05, 1.58) | 0.036* | -0.03 | (-0.70, 0.65) | 0.936 |
| Energy (kJ/d) | 0.00 | (-0.00, 0.00) | 0.906 | 0.00 | (-0.00, 0.00) | 0.552 |
| Sugar intake (g/d) | -0.01 | (-0.03, 0.02) | 0.072 | 0.01 | (-0.01, 0.03) | 0.181 |
| Healthy diet score | -0.59 | (-0.12, 0.02) | 0.061 | 0.23 | (-0.85, 0.23) | 0.264 |

Model was adjusted for age, sex, deprivation Index, education, alcohol intake, smoking status, hypertension, physical activity, laboratory measurements (glucose, triglyceride, cholesterol, C-reactive protein and platelet distribution width), dietary intake (total energy, total sugar, and healthy diet score) body mass index and abdominal obesity.

Table S13. Estimated direct and indirect effect category of beverages intake on PDFF mediated by mediators.

| **Category of Beverages intake** | **Mediator** | **Difference (95%Cl)** | | | **Mediated,(%)** |
| --- | --- | --- | --- | --- | --- |
|  |  | **Total effect (95%Cl)** | **Direct (95%Cl)** | **Indirect (95%Cl)** |  |
| **ASB** | **BMI** | 0.16 (0.11, 0.21) | 0.08 (0.03, 0.13) | 0.08 (0.07, 0.09) | 50.4% |
| **SSB** |  | 0.15 (0.10, 0.20) | 0.12 (0.07, 0.17) | 0.03 (0.01, 0.04) | 17.6% |
| **ASB** | **Sugar intake** | 0.09 (0.03, 0.14) | 0.08 (0.03, 0.14) | 0.00 (-0.00, 0.00) | Unmediated |
| **SSB** |  | 0.10 (0.04, 0.15) | 0.06 (0.01, 0.12) | 0.03 (0.02, 0.05) | 30.7% |
| **ASB** | **Healthy Diet Score** | 0.07 (0.03, 0.12) | 0.07 (0.02, 0.12) | 0.00 (0.00, 0.01) | 5.5% |
| **SSB** |  | 0.13 (0.09, 0.17) | 0.13 (0.07, 0.17) | 0.02 (0.02, 0.02) | 12.0% |
| **ASB** | **Platelet Distribution Width** | 0.07 (0.02, 0.12) | 0.07 (0.02, 0.12) | 0.00 (0.00, 0.01) | 3.2% |
| **SSB** |  | 0.12 (0.07, 0.16) | 0.12 (0.07, 0.16) | 0.00 (-0.00, 0.01) | Unmediated |
| **ASB** | **C-Reactive**  **Protein** | 0.07 (0.02, 0.12) | 0.06 (0.02, 0.12) | 0.01 (0.00, 0.01) | 5.8% |
| **SSB** |  | 0.12 (0.07, 0.16) | 0.11 (0.07, 0.16) | 0.01 (0.00, 0.00) | 4.3% |
| **ASB** | **Glucose** | 0.07 (0.02, 0.11) | 0.07 (0.02, 0.11) | -0.00 (-0.00, 0.00) | Unmediated |
| **SSB** |  | 0.11 (0.07, 0.16) | 0.11 (0.07, 0.16) | 0.00 (-0.00, 0.00) | Unmediated |
| **ASB** | **Cholesterol** | 0.16 (0.02, 0.11) | 0.16 (0.02, 0.11) | -0.00 (-0.00, 0.00) | Unmediated |
| **SSB** |  | 0.11 (0.07, 0.16) | 0.11 (0.07, 0.16) | -0.00 (-0.00, 0.00) | Unmediated |
| **ASB** | **Triglycerides** | 0.06 (0.02, 0.12) | 0.06 (0.02, 0.12) | 0.00 (-0.00, 0.01) | Unmediated |
| **SSB** |  | 0.13 (0.08, 0.17) | 0.11 (0.07, 0.15) | 0.02 (0.02, 0.03) | 12.0% |
| **ASB** | **Periodontitis** | 0.07 (0.03, 0.09) | 0.07 (0.03, 0.09) | 0.00 (0.00, 0.00) | 1.1% |
| **SSB** |  | 0.08 (0.04, 0.14) | 0.08 (0.04, 0.14) | 0.00 (-0.00, 0.00) | Unmediated |

Abbreviations: ASB:artificially-sweetened beverage; SSB: sugar-sweetened beverages; NJs:nature juices. Model was adjusted for age, sex, deprivation Index, education, alcohol intake, smoking status, hypertension, physical activity, laboratory measurements (glucose, triglyceride, cholesterol, C-reactive protein and platelet distribution width), dietary intake (total energy, total sugar, and healthy diet score) body mass index and abdominal obesity.

Table S14. Estimated direct and indirect effect category of beverages intake on CT1 mediated by mediators.

| **Category of Beverages intake** | **Mediator** | **Difference (95%Cl)** | | | **Mediated,(%)** |
| --- | --- | --- | --- | --- | --- |
|  |  | **Total effect (95%Cl)** | **Direct (95%Cl)** | **Indirect (95%Cl)** |  |
| **ASB** | **BMI** | 2.74 (1.82, 3.67) | 1.51 (1.29, 1.77) | 1.23 (0.32, 2.14) | 44.8% |
| **SSB** |  | 1.30 (0.52, 2.07) | 1.05 (0.29, 1.83) | 0.24 (0.11, 0.38) | 18.6% |
| **ASB** | **Sugar intake** | 1.73 (0.81, 2.65) | 1.67 (0.75, 2.59) | 0.05 (-0.01, 0.11) | Unmediated |
| **SSB** |  | 1.62 (0.87, 2.37) | 1.05 (0.29, 1.83) | 0.56 (0.39, 0.74) | 34.6% |
| **ASB** | **Healthy Diet Score** | 1.28 (0.42, 2.14) | 1.25 (0.39, 2.11) | 0.03 (0.01, 0.06) | 2.4% |
| **SSB** |  | 1.17 (0.41, 1.93) | 1.05 (0.29, 1.83) | 0.11 (0.05, 0.18) | 9.8% |
| **ASB** | **Platelet Distribution Width** | 1.25 (0.39, 2.11) | 1.24 (0.38, 2.10) | 0.02 (0.01, 0.02) | 7.7% |
| **SSB** |  | 1.06 (0.29, 1.83) | 1.06 (0.29, 1.82) | 0.00 (-0.02, 0.02) | Unmediated |
| **ASB** | **C-Reactive**  **Protein** | 1.25 (0.40, 2.11) | 1.15 (0.29, 2.01) | 0.10 (0.02, 0.19) | 8.1% |
| **SSB** |  | 1.05 (0.29, 1.82) | 0.97 (0.20, 1.73) | 0.09 (0.02, 0.16) | 8.4% |
| **ASB** | **Glucose** | 1.26 (0.39, 2.11) | 1.26 (0.39, 2.11) | -0.00 (-0.01, 0.01) | Unmediated |
| **SSB** |  | 1.06 (0.29, 1.83) | 1.06 (0.29, 1.83) | 0.00 (-0.00, 0.00) | Unmediated |
| **ASB** | **Cholesterol** | 1.38 (0.52, 2.24) | 1.25 (0.40, 2.12) | 0.13 (0.06, 0.21) | Unmediated |
| **SSB** |  | 1.06 (0.30, 1.83) | 1.06 (0.29, 1.82) | 0.00 (-0.05, 0.06) | Unmediated |
| **ASB** | **Triglycerides** | 1.23 (0.36, 2.10) | 1.26 (0.40, 2.11) | -0.02 (-0.15, 0.11) | Unmediated |
| **SSB** |  | 1.25 (0.48, 2.03) | 1.05 (0.29, 1.82) | 0.19 (0.07, 0.32) | 15.6% |
| **ASB** | **Periodontitis** | 1.25 (0.39, 2.12) | 1.25 (0.39, 2.11) | 0.01 (0.00, 0.01) | 1.0% |
| **SSB** |  | 1.05 (0.29, 1.82) | 1.05 (0.29, 1.83) | 0.01 (-0.01, 0.01) | Unmediated |

Abbreviations: ASB:artificially-sweetened beverage; SSB: sugar-sweetened beverages; NJs:nature juices. Model was adjusted for age, sex, deprivation Index, education, alcohol intake, smoking status, hypertension, physical activity, laboratory measurements (glucose, triglyceride, cholesterol, C-reactive protein and platelet distribution width), dietary intake (total energy, total sugar, and healthy diet score) body mass index and abdominal obesity.

Table S15. Sensitivity analyses of association between category of beverages intake and PDFF after adjusting additional covariate.

| **Category of Beverages intake** | **Model 5** | | **Model 6** | | **Model 7** | | **Model 8** | |
| --- | --- | --- | --- | --- | --- | --- | --- | --- |
|  | **Difference (95%Cl)** | **P** | **Difference (95%Cl)** | **P** | **Difference (95%Cl)** | **P** | **Difference (95%Cl)** | **P** |
| **Artificially-sweetened Beverages** | |  |  |  |  |  |  |  |
| 0 serving/d | reference |  | reference |  | reference |  | reference |  |
| 0-1 serving/d | -0.11 (-0.33, 0.12) | 0.342 | -0.05 (-0.25, 0.16) | 0.613 | -0.05 (-0.25, 0.15) | 0.602 | 0.11 (0.00, 0.23) | 0.054 |
| ≥1 serving/d | 0.12 (0.03, 0.22) | 0.013 | 0.13 (0.05, 0.22) | 0.003 | 0.13 (0.05, 0.22) | 0.003 | 0.21 (0.07, 0.35) | 0.002 |
| Per 1 serving/d increased | 0.07 (0.02, 0.13) | 0.005 | 0.08 (0.03, 0.12) | 0.001 | 0.07 (0.03, 0.12) | 0.002 | 0.09 (0.04, 0.14) | <0.001 |
| **Sugar-sweetened Beverages** | |  |  |  |  |  |  |  |
| 0 serving/d | reference |  | reference |  | reference |  | reference |  |
| 0-1 serving/d | 0.10 (0.05, 0.21) | 0.010 | 0.20 (0.06, 0.34) | 0.005 | 0.20 (0.06, 0.34) | 0.005 | 0.17 (0.08, 0.26) | <0.001 |
| ≥1 serving/d | 0.19 (0.10, 0.27) | <0.001 | 0.21 (0.13, 0.29) | <0.001 | 0.21 (0.13, 0.29) | <0.001 | 0.30 (0.17, 0.42) | <0.001 |
| Per 1 serving/d increased | 0.10 (0.05, 0.16) | <0.001 | 0.13 (0.08, 0.17) | <0.001 | 0.12 (0.08, 0.16) | <0.001 | 0.11 (0.06, 0.17) | <0.001 |
| **Nature juices** | |  |  |  |  |  |  |  |
| 0 serving/d | reference |  | reference |  |  |  |  |  |
| 0-1 serving/d | -0.08 (-0.18, -0.05) | 0.029 | -0.09 (-0.17, -0.00) | 0.038 | -0.09 (-0.17, -0.00) | 0.035 | -0.02 (-0.12, 0.07) | 0.597 |
| ≥1 serving/d | 0.00 (-0.08, 0.10) | 0.919 | 0.02 (-0.06, 0.10) | 0.585 | 0.02 (-0.06, 0.10) | 0.596 | -0.07 (-0.23, 0.09) | 0.365 |
| Per 1 serving/d increased | 0.03 (-0.04, 0.09) | 0.452 | 0.03 (-0.03, 0.09) | 0.362 | 0.03 (-0.03, 0.09) | 0.31 | 0.02 (-0.05, 0.09) | 0.579 |

Model 5 was adjusted for original seven parts of healthy diet score rather than healthy diet score.

Model 6 was additionally adjusted for carbohydrate intake.

Model 7 was additionally adjusted for use of medication (aspirin and lowing cholesterol medicine).

Model 8 was built in those who completed at least 2 dietary questionnaires (n=17,607)

Table S16. Sensitivity analyses of association between category of beverages intake and CT1 after adjusting additional covariate.

| **Category of Beverages intake** | **Model 5** | | **Model 6** | | **Model 7** | | **Model 8** | |
| --- | --- | --- | --- | --- | --- | --- | --- | --- |
|  | **Difference (95%Cl)** | **P** | **Difference (95%Cl)** | **P** | **Difference (95%Cl)** | **P** | **Difference (95%Cl)** | **P** |
| **Artificially-sweetened Beverages** | |  |  |  |  |  |  |  |
| 0 serving/d | reference |  | reference |  | reference |  | reference |  |
| 0-1 serving/d | 3.34 (1.25, 7.93) | 0.155 | 3.86 (-0.25, 7.97) | 0.066 | 3.63 (-0.47, 7.74) | 0.083 | 3.27 (-1.52, 7.70) | 0.147 |
| ≥1 serving/d | 2.18 (0.27, 4.09) | 0.025 | 2.45 (0.72, 4.17) | 0.005 | 2.37 (0.64, 4.09) | 0.007 | 2.52 (0.56, 4.48) | 0.012 |
| Per 1 serving/d increased | 1.77 (0.75, 2.79) | <0.001 | 1.64 (0.72, 2.56) | <0.001 | 1.64 (0.72, 2.56) | <0.001 | 1.65 (0.60, 2.70) | 0.002 |
| **Sugar-sweetened Beverages** | |  |  |  |  |  |  |  |
| 0 serving/d | reference |  | reference |  | reference |  | reference |  |
| 0-1 serving/d | 1.52 (0.09, 3.01) | 0.021 | 1.90 (0.35, 3.44) | 0.016 | 1.26 (0.54, 3.99) | 0.002 | 1.73 (0.17, 3.86) | 0.022 |
| ≥1 serving/d | 1.81 (0.11, 3.52) | 0.037 | 2.37 (0.65, 4.10) | 0.002 | 1.89 (0.34, 3.43) | 0.017 | 2.01 (0.25, 3.78) | 0.025 |
| Per 1 serving/d increased | 0.94 (0.05, 1.84) | 0.044 | 0.85 (0.04, 1.85) | 0.033 | 0.88 (0.11, 1.78) | 0.043 | 1.13 (0.34, 1.71) | 0.003 |
| **Nature juices** | |  |  |  |  |  |  |  |
| 0 serving/d | reference |  | reference |  | reference |  | reference |  |
| 0-1 serving/d | -0.19 (-2.07, 1.68) | 0.838 | -0.18 (-1.89, 1.52) | 0.830 | -0.23 (-1.94, 1.47) | 0.790 | 0.90 (-1.07, 2.87) | 0.371 |
| ≥1 serving/d | 1.34 (-0.43, 3.12) | 0.139 | 0.80 (-0.80, 2.41) | 0.329 | 0.71 (-0.89, 2.32) | 0.386 | 1.61 (-0.31, 3.53) | 0.100 |
| Per 1 serving/d increased | 1.37 (-0.05, 2.68) | 0.141 | 0.97 (-0.20, 2.15) | 0.105 | 0.92 (-0.25, 2.10) | 0.126 | 1.55 (-0.16, 2.94) | 0.128 |

Model 5 was adjusted for original seven parts of healthy diet score rather than healthy diet score.

Model 6 was additionally adjusted for carbohydrate intake.

Model 7 was additionally adjusted for use of medication (aspirin and lowing cholesterol medicine).

Model 8 was built in those who completed at least 2 dietary questionnaires (n=17,607)

Table S17. Sensitivity analyses of association between category of beverages intake with PDFF and CT1 in population with least 2 dietary questionnaires (n=17,607).

| **Category of Beverages intake** | **PDFF** | | **CT1** | |
| --- | --- | --- | --- | --- |
|  | **Difference (95%Cl)** | **P** | **Difference (95%Cl)** | **P** |
| **Artificially-sweetened Beverages** |  |  |  |  |
| 0 serving/d | reference |  | reference |  |
| 0-1 serving/d | 0.11 (0.00, 0.23) | 0.054 | 3.27 (-1.52, 7.70) | 0.147 |
| ≥1 serving/d | 0.21 (0.07, 0.35) | 0.002 | 2.52 (0.56, 4.48) | 0.012 |
| Per 1 serving/d increased | 0.09 (0.04, 0.14) | <0.001 | 1.65 (0.60, 2.70) | 0.002 |
| **Sugar-sweetened Beverages** |  |  |  |  |
| 0 serving/d | reference |  | reference |  |
| 0-1 serving/d | 0.17 (0.08, 0.26) | <0.001 | 1.73 (0.17, 3.86) | 0.022 |
| ≥1 serving/d | 0.30 (0.17, 0.42) | <0.001 | 2.01 (0.25, 3.78) | 0.025 |
| Per 1 serving/d increased | 0.11 (0.06, 0.17) | <0.001 | 1.13 (0.34, 1.71) | 0.003 |
| **Nature juices** |  |  |  |  |
| 0 serving/d |  |  | reference |  |
| 0-1 serving/d | -0.02 (-0.12, 0.07) | 0.597 | 0.90 (-1.07, 2.87) | 0.371 |
| ≥1 serving/d | -0.07 (-0.23, 0.09) | 0.365 | 1.61 (-0.31, 3.53) | 0.100 |
| Per 1 serving/d increased | 0.02 (-0.05, 0.09) | 0.579 | 1.55 (-0.16, 2.94) | 0.128 |

Model was adjusted for age, sex, deprivation Index, education, alcohol intake, smoking status, hypertension, physical activity, laboratory measurements (glucose, triglyceride, cholesterol, C-reactive protein and platelet distribution width), dietary intake (total energy, total sugar, and healthy diet score) body mass index and abdominal obesity.

Table S18. Sensitivity analyses of the association between category of beverage intake and percentage change of PDFF.

| **Category of Beverage intake** | **Model 1** | | **Model 2** | | **Model 3** | | **Model 4** | |
| --- | --- | --- | --- | --- | --- | --- | --- | --- |
|  | **Difference (95%Cl)** | **P** | **Difference (95%Cl)** | **P** | **Difference (95%Cl)** | **P** | **Difference (95%Cl)** | **P** |
| **Artificially-sweetened Beverages** | |  |  |  |  |  |  |  |
| 0 serving/d | reference |  | reference |  | reference |  | reference |  |
| 0-1 serving/d | 0.92 (0.61, 1.23) | <0.001 | 1.24 (0.93, 1.55) | <0.001 | 0.88 (0.61, 0.52) | <0.001 | -1.26 (-2.83, 0.52) | 0.184 |
| ≥1 serving/d | 1.48 (1.12, 1.85) | <0.001 | 1.94 (1.58, 2.30) | <0.001 | 1.38 (1.05, 1.70) | <0.001 | 1.39 (0.08, 0.70) | 0.014 |
| Per 1 serving/d increased | 2.66 (2.18, 3.13) | <0.001 | 3.33 (2.86, 3.13) | <0.001 | 2.26 (1.75, 2.13) | <0.001 | 0.56 (0.14, 0.13) | 0.008 |
| **Sugar-sweetened Beverages** | |  |  |  |  |  |  |  |
| 0 serving/d | reference |  | reference |  | reference |  | reference |  |
| 0-1 serving/d | 2.12 (1.12, 3.16) | <0.001 | 2.12 (1.16, 3.12) | <0.001 | 1.96 (1.08, 2.88) | <0.001 | 1.89 (0.66, 3.12) | 0.003 |
| ≥1 serving/d | 1.42 (1.08, 1.75) | <0.001 | 1.16 (0.84, 1.49) | <0.001 | 1.07 (0.76, 1.38) | <0.001 | 1.80 (1.11, 2.50) | <0.001 |
| Per 1 serving/d increased | 2.19 (1.73, 2.13) | <0.001 | 1.80 (1.34, 2.13) | <0.001 | 1.63 (1.20, 2.13) | <0.001 | 1.08 (0.67, 1.13) | <0.001 |
| **Nature juices** | |  |  |  |  |  |  |  |
| 0 serving/d | reference |  | reference |  | reference |  | reference |  |
| 0-1 serving/d | -0.44 (-0.67, 0.20) | <0.001 | -0.63 (-0.85, -0.40) | <0.001 | -0.29 (-0.49, -0.08) | 0.007 | -0.95 (-1.73, -0.17) | 0.016 |
| ≥1 serving/d | 0.03 (-0.35, 0.42) | 0.869 | -0.40 (-0.78, -0.03) | 0.034 | -0.13 (-0.49, 0.23) | 0.475 | -0.10 (-0.62, 0.82) | 0.786 |
| Per 1 serving/d increased | -0.48 (-1.10, 0.12) | 0.119 | -1.22 (-1.81, -0.63) | <0.001 | -0.07 (-0.63, 0.50) | 0.818 | 0.18 (-0.35, 0.71) | 0.540 |

Model 1:Unadjusted.

Model 2: Adjusted for age and sex.

Model 3: Model 2 plus deprivation Index, education, alcohol intake, smoking status, hypertension, physical activity, laboratory measurements (glucose, triglyceride, cholesterol, C-reactive protein and platelet distribution width), dietary intake (total energy, total sugar, and healthy diet score).

Model 4: Model 3 plus body mass index and abdominal obesity.

Figure S1: schematic summarizing of substitution effects.


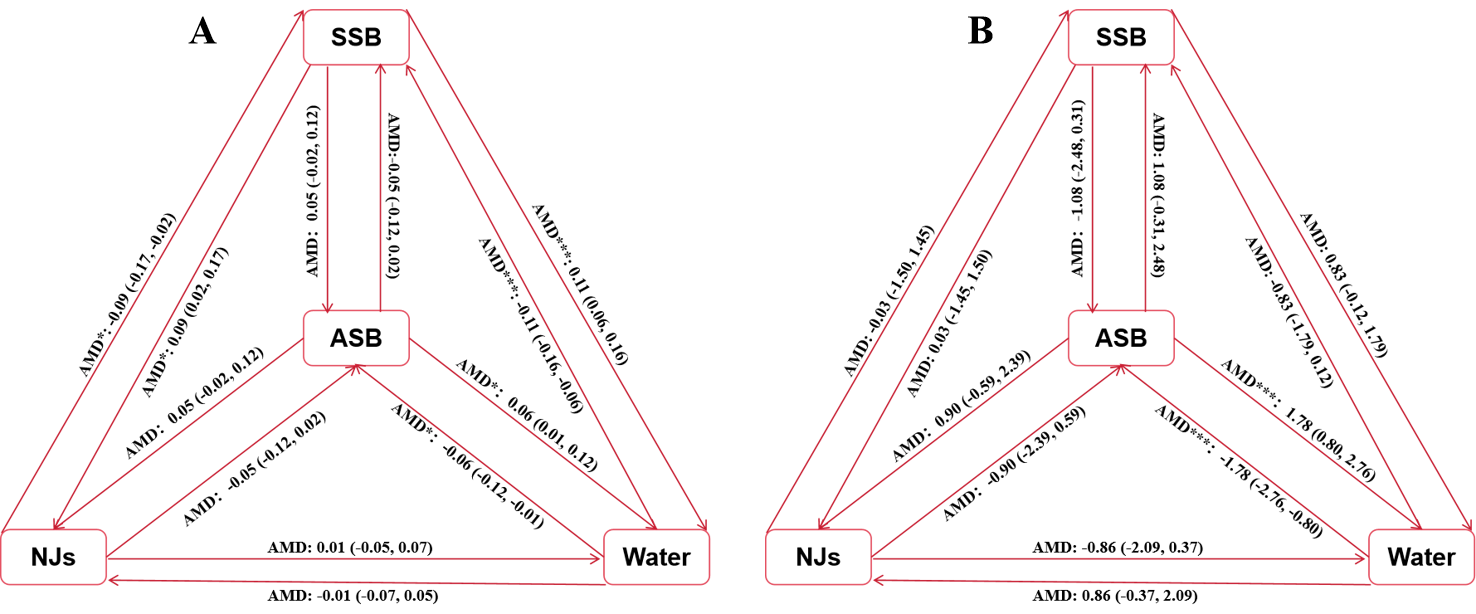


Substitution analysis examining the association between PDFF (A) as well as cT1 (B) and category of beverage intake.

AMD: arithmetic mean difference;

*0.01<p<0.05, **0.001<p<0.01, ***p:<0.001;

ASB→SSB：Replace SSB with ASB.
